# A Pathogen-Responsive Leucine Rich Receptor Like Kinase Contributes to *Fusarium* Resistance in Cereals

**DOI:** 10.3389/fpls.2018.00867

**Published:** 2018-06-26

**Authors:** Ganesh Thapa, Lokanadha R. Gunupuru, James G. Hehir, Amal Kahla, Ewen Mullins, Fiona M. Doohan

**Affiliations:** ^1^UCD School of Biology and Environmental Science, UCD Earth Institute and UCD Institute of Food and Health, University College of Dublin, Belfield, Ireland; ^2^Crop Science Department, Oak Park Crops Research Centre, Teagasc, Carlow, Ireland

**Keywords:** Leucine rich repeat receptor like kinase (LRR-RLK), *Triticum aestivum*, *Fusarium*, Pathogen-associated molecular pattern (PAMP), Virus-induced gene silencing (VIGS)

## Abstract

Receptor-like kinases form the largest family of receptors in plants and play an important role in recognizing pathogen-associated molecular patterns and modulating the plant immune responses to invasive fungi, including cereal defenses against fungal diseases. But hitherto, none have been shown to modulate the wheat response to the economically important Fusarium head blight (FHB) disease of small-grain cereals. Homologous genes were identified on barley chromosome 6H (*HvLRRK-6H*) and wheat chromosome 6DL (*TaLRRK-6D*), which encode the characteristic domains of surface-localized receptor like kinases. Gene expression studies validated that the wheat *TaLRRK-6D* is highly induced in heads as an early response to both the causal pathogen of FHB disease, *Fusarium graminearum*, and its’ mycotoxic virulence factor deoxynivalenol. The transcription of other wheat homeologs of this gene, located on chromosomes 6A and 6B, was also up-regulated in response to *F. graminearum*. Virus-induced gene silencing (VIGS) of the barley *HvLRRK-6H* compromised leaf defense against *F. graminearum*. VIGS of *TaLRRK-6D* in two wheat cultivars, CM82036 (resistant to FHB disease) and cv. Remus (susceptible to FHB), confirmed that *TaLRRK-6D* contributes to basal resistance to FHB disease in both genotypes. Although the effect of VIGS did not generally reduce grain losses due to FHB, this experiment did reveal that *TaLRRK-6D* positively contributes to grain development. Further gene expression studies in wheat cv. Remus indicated that VIGS of *TaLRRK-6D* suppressed the expression of genes involved in salicylic acid signaling, which is a key hormonal pathway involved in defense. Thus, this study provides the first evidence of receptor like kinases as an important component of cereal defense against *Fusarium* and highlights this gene as a target for enhancing cereal resistance to FHB disease.

## Introduction

Plants have evolved specialized mechanisms to defend themselves against invading microbial pathogens. Defense signaling cascades are induced upon the recognition of pathogen-associated molecular patterns (PAMPs) by pattern recognition receptors (PRRs) (Cohn et al., 2001; Dangl and Jones, 2001; Jones and Dangl, 2006). PRRs are either surface-localized receptor-like kinases (RLKs) or receptor-like proteins (RLPs). RLKs comprise a ligand-binding ectodomain, transmembrane domain, and an intracellular kinase domain, while RLPs contains both an ecto-ligand binding and transmembrane domain with only a short cytoplasmic domain that lacks an obvious signaling intracellular kinase domain (Tör et al., 2009; Sanabria et al., 2010; Antolín-Llovera et al., 2012; Macho and Zipfel, 2014). Surface-localized PRRs lead to PRR-triggered immunity. This is complemented by a second layer of intracellular resistance driven by NOD-like receptor (NLR) PRRs, which recognize virulence effectors secreted within host cells by pathogens, thereby inducing effector-triggered immunity (ETI) (Boller and Felix, 2009; Cui et al., 2015; Delga et al., 2015).

RLKs with leucine-rich repeat (LRR) ectodomains form the largest family of receptors in plants (Torii, 2004; Matsushima and Miyashita, 2012; Fischer et al., 2016). The classic LRR-RLK example is the *Arabidopsis* flagellin sensing 2 (FLS2), which binds bacterial flagellin (flg22) (Gómez-Gómez and Boller, 2000; Chinchilla et al., 2006). LRR-RLKs can act together in mediating the trade-off between growth and immunity (Belkhadir et al., 2014; Lozano-Durán and Zipfel, 2015). For example, the LRR-RLK brassinosteroid-insensitive 1 (BRI1) -associated kinase (BAK1) forms a complex with FLS2 in order to bind the plant growth regulating brassinosteroid hormones, but BAK1 also complexes with FLS2 to initiate PAMP-triggered immunity (Gómez-Gómez and Boller, 2000; Vert et al., 2005; Chinchilla et al., 2007; Boller and Felix, 2009; Chinchilla et al., 2009; Sun et al., 2013; Wu and Zhou, 2013). The LRR-RLK BAK1-interacting receptor-like kinase (BIR2) was reported to interact with BAK1 in the absence of PAMPs to inhibit autoimmune cell-death responses, thus keeping BAK1 under control. Only upon ligand binding to FLS2 is BAK1 released from BIR2 and recruited to the FLS2 complex to induce PAMP-triggered immune signaling (Halter et al., 2014). Overexpression of *Arabidopsis* BAK1 led to increased accumulation of salicylic acid (SA) and the deregulation of cell death control genes (Kim et al., 2017).

Fungal chitin is an important ligand for LRR-RLKs. The rice lysine-motif (LysM) receptor kinase has been reported to recognize the fungal elicitor chitin (Kaku et al., 2006) and the *Arabidopsis* receptor kinase CERK1 binds fungal chitin (Miya et al., 2007). Upon binding chitin, there is a rapid phosphorylation of CERK1 and the activation of early defense responses (Petutschnig et al., 2010). Similarly, CEBiP (chitin elicitor binding protein), which functions in cooperation with CERK1, also activates defense signaling against the rice blast fungus *Magnaporthe oryzae* and the pathogen overcomes this first line of defense by secreting an effector protein, Secreted LysM Protein1 (Slp1), to cause the disease (Chen et al., 2014). In cereal crops, there are several recent examples of RLKs playing a role in the defense against fungal pathogens. CEBiP and CERK1 play a role in the defense against Septoria tritici blotch (STB) disease; a mutant of the causal pathogen disrupted in the effector Mg3LysM [chitin-binding lysin motif (LysM) containing fungal effector homologue of Ecp6] is normally non-pathogenic, but virus-induced gene silencing (VIGS) of the *CEBiP* and *CERK1* genes enabled the mutant to colonize leaf tissue (Lee et al., 2014, 2015). A recent study demonstrated that the silencing of barley *HvCERK1* compromised resistance to Fusarium head blight (FHB) disease and complementary gene expression and metabolomics studies elucidated its impact on downstream resistance-related metabolite accumulation (Karre et al., 2017). Semidwarf “uzu” barley lines encode a spontaneous mutation in the kinase domain of BRI1, which renders them more resistant to the economically important FHB disease and stem base disease caused by *Fusarium* fungi (Ali et al., 2014). Gene expression studies were used to determine that the uzu derivatives are attenuated in downstream brassinosteroid signaling. The reduction of *BRI1* RNA levels via VIGS compromised uzu disease resistance, suggesting that mutated BRI1 in uzu is still in some way functional and that the altered function confers uzu lines with the enhanced disease resistance. The authors concluded that the pathogen resistance of uzu derivatives might be due to pleiotropic effects of BRI1 or the cascade effects of their repressed BR signaling.

A gene expression study highlighted genes involved in the uzu barley response to *Fusarium* inoculation at both 24 h (Ali et al., 2014) and 48 h post-pathogen inoculaton (Ali et al., unpubl. data). The expression of an uncharacterised *LRR-RLK* gene was seven-fold higher in spikelets of “uzu” versus wild type barley in response to inoculation with *Fusarium culmorum* at 48 h post-fungal treatment. Herein, we characterize this barley *LRR-RLK* gene and its *Fusarium-*responsive wheat homeologs and their role in cereal resistance to the FHB causal agent *Fusarium graminearum*. FHB is a devastating disease of wheat that causes yield loss and contaminates grain with the mycotoxin deoxynivalenol (DON). In addition to being harmful to human and animal health, DON is also a fungal virulence factor, aiding pathogen colonization of the wheat tissue (Proctor et al., 1995). Using gene expression analysis, we studied the responsive of the wheat homeologs to DON and DON-producing *F. graminearum*. Using VIGS, we determined the contribution of the *Fusarium-*responsive barley and wheat *LRR-RLK* genes to host resistance to FHB disease and associated yield loss. SA is key hormonal pathway activated as an early response to FHB disease (Makandar et al., 2012; Sorahinobar et al., 2016) and gene expression studies analyzed the effect of *LRR-RLK* gene silencing in cv. Remus on the transcription of key genes involved in SA accumulation, perception and signaling. Our results highlight this LRR-RLK as a contributor to basal defense against FHB disease and point to its importance as an upstream component of SA signaling in wheat.

## Materials and Materials

### Plant Material, Growth Conditions and Fungal Treatment

Wheat cultivars (cvs.) CM82036 [resistant to both FHB disease and DON; (Buerstmayr et al., 2003)], Remus [susceptible to FHB; (Buerstmayr et al., 1996)], Chinese Spring and its derivative nullisomic tetrasomic lines (obtained from Germplasm Resources Unit, JIC, Norwich^1^) (Supplementary Table S1) were grown under contained environment conditions, as previously described (Ansari et al., 2007). The barley cv. Akashinriki is susceptible to FHB, while its uzu derivative is more resistant (Khan and Doohan, 2009; Ali et al., 2014). Fresh asexual conidial inoculum (macroconidia) of *F. graminearum* wild type GZ3639 (Bai et al., 2002) and its’ trichothecene-minus mutant derivative GZT40 (Proctor et al., 1995) and *F. culmorum* strain FCF200 (kindly provided by Dr. Paul Nicholson, John Innes Centre, Norwich, United Kingdom) were cultured on potato dextrose agar (PDA) (Difco, United Kingdom) plates, incubated at 25°C for 5 days. For conidial production, fungi were cultured in mung bean broth (Bai and Shaner, 1996), harvested, washed and adjusted to 10^6^ conidia ml^-1^, as previously described (Brennan et al., 2005).

### DNA, RNA Extraction and cDNA Synthesis

DNA was extracted from wheat spikelets and barley leaves using the HP plant DNA mini kit (OMEGA) following the manufacturer’s instructions. RNA was extracted from wheat heads and barley leaves as previously described (Ansari et al., 2007) and was DNase-treated using the TURBO DNA-*free* TM kit (Ambion Inc., United States). The quality, yield and integrity of the RNA was analyzed using both the ND-1000 spectrophotometer (NanoDrop, Thermo Fisher Scientific, United States) and electrophoresis. Reverse transcription of total RNA and the quality check of synthesized cDNA for DNA contamination was conducted as previously described (Walter et al., 2008).

### Cloning of the *TaLRRK-6D* Gene

The barley LRR-RLK (MLOC_12033.1) on chromosome 6H (Matsumoto et al., 2011) was used as a model for gene cloning. We identified a wheat homolog on chromosome 6DL of wheat cv. Chinese Spring (TRIAE_CS42_6DL_TGACv1_-527217_AA1700660.1) via BLASTn analysis of the wheat genome sequence.^2^ This gene is hereafter referred to as *TaLRRK-6D*. *TaLRRK-6D* from wheat cvs. CM82036 and Remus was cloned and sequenced from mRNA following several rounds of 5′/3′ RACE, using gene-specific primers designed along the coding sequence of *TaLRRK-6D* (Supplementary Table S2). PCR reactions (25 μl volume) contained 0.5 μl of cDNA template, 2 μM each of gene-specific reverse/forward primer and either 5′ GeneRacer TM primer or 5′ nested/3′ forward GeneRacer TM primer, 1.25 U of Takara LA Taq TM and 1X LA buffer II (Mg^2+^ plus) (Takara Bio Inc., Japan), and 0.4 mM of each dNTP. Reaction conditions were as follows: 94°C for 2 min, 30 cycles of 94°C for 30 s and 68°C for 3 min and a final extension step at 72°C for 10 min and were conducted in a ProFlex PCR System (Applied Biosystems by Life Technologies, United States). The amplified PCR products were cloned into the pGEM-T vector (pGEM-XL Easy cloning kit; Promega, United Kingdom) and sequenced using both gene-specific and plasmid-specific primers (Supplementary Table S2). The reads were aligned with the deduced full-length gene sequences, which were confirmed to be on chromosome 6DL based on BLASTn analysis against the wheat genome.^3^

### Sequence and Phylogenetic Analysis

*TaLRRK-6D* sequences from cvs. CM82036 and Remus were used to extract homologs via BLASTp against the EnsemblPlants database,^3^ *Triticum aestivum* (TGACv1) (Kersey et al., 2016) and Gramene (Monaco et al., 2014). These sequences were MAFFT-aligned (Katoh et al., 2002) using Blosum62 matrix (Eddy, 2004), a gap opening penalty of 1.53 and an offset value of 0.123. Then the mean pairwise identity values were used to generate a phylogenetic tree using Jukes Cantor genetic distant model and the Neighbor joining tree building method within the Geneious Tree builder^4^ (Geneious R9 v.9.1.3) (Kearse et al., 2012). For domain analysis, the amino-acid sequence of *TaLRRK-6D* was analysis via BLASTp against the plant.ensembl.org and integrated InterPro protein database of all annotated eukaryotic genes in Geneious R9 v.9.1.3. LRR-RLKs were further scanned in Geneious R9 v.9.1.3 for the presence of signature domains, transmembrane domains and signal peptide using SMART^5^ (Letunic et al., 2015), PROSITE analysis^6^ (Sigrist et al., 2012), HAMAP^7^ (Pedruzzi et al., 2015), PRINTS^8^ (Attwood et al., 2012), PFAM^9^ (Finn et al., 2013), SUPERFAMILY^10^ (Wilson et al., 2009) and InterPro^11^ (Mitchell et al., 2014). Signalp v4.1 (Petersen et al., 2011), TMHMM v2.0 (Krogh et al., 2001) and Phobius (Käll et al., 2007).

### Wheat Adult Plant Time Course FHB and DON Treatment Experiments

An adult plant experiment was conducted to analyze the temporal response of *TaLRRK-6D* and its homeologs to both DON and FHB disease in the wheat cv. CM82036. The wheat plants were grown under contained glasshouse environment conditions, two plants per pot, as previously described (Ansari et al., 2007), with minor modifications. At mid anthesis (growth stage 65) (Zadoks et al., 1974), the two central spikelets of wheat heads were treated with 20 μl (40 μl per head) of either DON (Santa Cruz, Texas, United States) (5 mg ml^-1^ in 0.02% Tween-20,), or 10^6^ conidia ml^-1^ of either wild type *F. graminearum* strain GZ3639 (WT) or its DON-minus derivative GZT40 which is mutated in the key mycotoxin biosynthesis gene *Tri5* (Proctor et al., 1995) or 0.02% Tween-20 (mock treatment). One head was treated per plant. After treatment, the heads were covered with a plastic bag for 2 days to maintain high humidity. Treated spikelets were harvested at either 0, 1, 2, 3, or 5 days post-treatment, flash-frozen in liquid N_2_ and stored at -70°C prior to RNA extraction. The experiment comprised a total of eight heads per treatment combination (two trials, each containing four heads per treatment combination). For gene expression studies, RNA was extracted from the two treated spikelets per head and, for each treatment combination, RNA samples were bulked to give a total of four RNA samples (two per trial).

### Preparation of the Virus-Induced Gene Silencing (VIGS) Constructs and Derivative RNA

The barley stripe mosaic virus (BSMV)-derived VIGS vectors used in this study consisted of the wild type BSMV ND18 α, β, and γ tripartite genome (Holzberg et al., 2002; Scofield et al., 2005). Two constructs were independently used for gene silencing. The two constructs, BSMV:LRR1 and BSMV:LRR2, were designed to preferentially target the kinase domains of barley LRR-RLK on chromosome 6H, its wheat homolog *TaLRRK-6D*, but not the wheat homologs on chromosome 6A, 6B, 2A, 2B, and 2D (Supplementary Table S3). Construct specificity was determined via a combination of (i) homology of at least 25 nt long with VIGS fragment to the *LRR-RLK* variant gene sequence in wheat of (cv. Chinese Spring, CS) or barley (cv. Morex), (ii) siRNA finder si-fi tool^12^ and (iii) quantitative reverse transcriptase PCR analysis (qRT-PCR) with variant-specific primers in wheat (cvs. CM82036 and Remus) or barley (cv. Akashinriki) (Table S3). Fragments were amplified from wheat cv. CM82036 cDNA of *TaLRRK-6D* using the VIGS primers (Supplementary Table S2; see qRT-PCR section below) and were ligated in the antisense orientation into NotI/Pac1-digested BSMV γ vector pSL038-1 (Scofield et al., 2005). The construct authenticity was verified by sequencing. A BSMV γ vector construct containing a 185 bp-fragment of the barley phytoene desaturase gene (BSMV:*PDS*) was used as positive control for VIGS, as previously described (Scofield et al., 2005). Prior to RNA synthesis, the vectors were linearized (the vectors containing the BSMV α and γ genomes and the γ genome vectors containing either BSMV:LRR1, BSMV:LRR2, or BSMV:*PDS* were linearized with *Mlu*I, while the BSMV β genome was linearized with *SpeI*). Capped *in vitro* transcripts were prepared from the linearized plasmids using the mMessage mMachine T7 *in vitro* transcription kit (AM1344, Ambion) following the manufacturer’s protocol. RNA quantity and quality were evaluated using ND-1000 spectrophotometer (NanoDrop, Thermo Fisher Scientific, United States) measurement and agarose (Sigma-Aldrich, United States, 1.5%) gel electrophoresis.

### VIGS Analysis in Barley and Wheat

Ali et al. (2014) reported the use of a detached leaf assay to analyze the response of barley to *F. culmorum.* This assay was used to assess the effect of VIGS of the barley LRR-RLK (MLOC_12033.1) on the response of leaves to *F. culmorum.* Perochon et al. (2015) reported the use of VIGS to silence genes in wheat heads and this assay was used to assess the effect of VIGS of wheat *TaLRRK6D* on the development of FHB disease caused by *F. graminearum* on wheat heads. In both the barley and wheat experiments, the VIGS treatment applied were either the VIGS buffer FES or this buffer containing a 1:1:1 mixture of the *in vitro* transcripts of BSMV α, β, and γ RNA (BSMV:00), or of BSMV α and β plus the γ RNA that contained the appropriate gene fragment (BSMV:*PDS*, BSMV:LRR1, or BSMV:LRR2) (Scofield et al., 2005).

For the detached leaf barley experiment, the second leaf of 10-day-old barley cv. Akashinriki or its uzu derivative plants were rub-inoculated with the VIGS treatment and, after 7 days, the third leaf of each treated plant was harvested and cut into three sections of 2, 3, and 3 cm in length. The 2 cm section was flash frozen for subsequent gene expression analysis (to determine the efficacy of gene silencing). The two 3 cm long leaf sections were used in the detached leaf phenotyping assay. The center of each leaf section was punctured with a glass Pasteur pipette and treated with a 5 μl droplet of either 0.02% Tween-20 (mock treatment) or conidia of *F. culmorum* strain FCF200 (10^6^ spores ml^-1^) as described earlier (Ali et al., 2014). The experiment included three trials, each of which included 10 plates per treatment with two leaf sections per plate. The plates were incubated at 22°C under a 16 h light/8 h dark cycle and all leaf sections were analyzed 4 days post-inoculation for both disease severity and macroconidial production (60 leaves analyzed per treatment combination). Diseased leaf area was estimated using IMAGE-J software analysis of the photographed leaf sections (Perochon and Doohan, 2016). Leaf segments were suspended in 2 ml of distilled water and vortexed and the macroconidial concentration in the water was determined using a haemocytometer (Hycor Biomedical, United States). For gene expression analysis for the barley VIGS leaf experiment, RNA was extracted from individual leaves and then equivalent amounts were bulked from the two leaves within the same plate, resulting in 30 bulk RNA samples per treatment combination (10 per trial).

For the wheat VIGS experiment, just before the emergence of heads of cvs. CM82036 and Remus, the flag leaf was rub-inoculated with the VIGS treatment. At mid-anthesis two central spikelets of heads on VIGS-treated tillers were treated with either 10^6^ conidia ml^-1^ of *F. graminearum* strain GZ3639 or of 0.02% Tween-20 (mock treatment) as described above in the time course experiment. After 24 h, the third spikelet above the treated spikelet was harvested, flash frozen in liquid N_2_ and stored at -70°C for subsequent gene expression analysis (to determine the efficacy of silencing; note that preliminary optimization experiments indicated that in cv. Remus the gene was significantly activated in this distal tissue at 1 dpi). Thereafter, the number of diseased (discolored) spikelets (including treated spikelets) was assessed at 7 and 14 dpi. At harvest, the number of seeds per head and average seed weight (mg) was determined. For disease and yield assessment, the experiment comprised three trials, each including 20 heads/biological replicates per treatment combination (five plants, two per pot and two heads per plant). For gene expression analysis, RNA was extracted from individual spikelets (each representing an independent head) from two of the trials and equivalent amounts of RNA representing the four treated heads per pot were bulked to give a total of ten bulk RNA samples per treatment combination (five per trial).

### Quantitative Reverse Transcriptase PCR

Real time qRT-PCR was conducted using the Mx3000p Real-Time PCR (Stratagene, Germany). Each PCR reaction contained 1.25 μl of 1:5 (vv^-1^) dilution of cDNA and 0.2 μM each of the forward and reverse primers (Supplementary Table S2), 1X SYBR^®^ Premix Ex Taq^TM^ (Tli RNase H plus, RR420A, Takara) in a total reaction volume of 12.5 μl. PCR reaction conditions were: 1 cycle of 1 min at 95°C; 40 cycles of 5 s at 95°C and 20 s at 58°C; a final cycle of 1 min at 95°C, 30 s at 58°C, and 30 s at 95°C for the dissociation curve. To analyze the temporal expression and VIGS of *TaLRRK-6D* and its variants via VIGS, primers were designed for each homeologue that were both variant-specific and targeted a region distinct from the VIGS targets; primers specific to *HvLRRK-6H* were used to analyze gene silencing via VIGS (Supplementary Table S2). The specificity of the primers was validated via sequence analysis of PCR product clones and by confirming the lack of PCR amplification in DNA from the relevant wheat cv. Chinese Spring nullisomic-tetrasomic lines. The wheat α-tubulin (GenBank no. U76558.1) (Xiang et al., 2011) and *TaGAPDH2* (Perochon et al., 2015) were used as housekeeping genes for all wheat qRT-PCR analysis (verified to be unaffected by either VIGS or *Fusarium* treatments). The barley actin (*HvActin*, Accession number: AY145451.1) (Ferdous et al., 2015) and *Hvα-tubulin* (Affymetrix Contig127_s_at) (Ali et al., 2014) were used as housekeeping genes for barley VIGS qRT-PCR analysis. To analyze the effect of silencing of *TaLRRK-6D* on SA signaling in wheat defense against FHB, primers were designed to amplify variants/homeologs of the SA biosynthesis genes *ICS1* and *PAL1*, the SA regulator *NPR1* and the SA receptors *NPR3-like* and *NPR4* (see Supplementary Table S4). Genes from *Arabidopsis* and Rice were used to identify wheat cDNA homologs via BLASTn in Ensembl Plants [*Triticum aestivum* (TGACv1)^13^. The *Fusarium* responsiveness and expression profile of target *ICS1*, *NPR1, NPR3-like*, and *NPR4* genes was analyzed using the Wheat Expression Browser,^14^ PLEXdb (Plant Expression Database), Expression Atlas^15^ and the SRA database.^16^ The bulked RNA sample numbers analyzed per treatment combination were: 4 (wheat time course experiment, representing 8 replicates), 30 (VIGS barley leafs, representing 60 replicates), and 10 (VIGS wheat heads for both validation of gene silencing and SA genes, representing 40 replicates). All qRT-PCR analyses were conducted in duplicate for each sample For plant gene expression studies, the threshold cycle (CT) values obtained by qRT-PCR were used to calculate the relative gene expression using the formula = [2^-(Ct target gene-Ct of the housekeeping gene1)^ + (2^-(Ct target gene-Ct of the housekeeping gene2)^]/2. The relative expression of the *ICS1*, *NPR1, NPR3-like*, and *NPR4* in samples was expressed as the fold change relative to FES (VIGS buffer) mock treatment and was calculated using the formula [(Etarget)^ΔCt target (control-sample)^/(Ehousekeeping) ^ΔCt housekeeping (control-sample)^] (Souaze et al., 1996).

### Statistical Analysis

The equality of the variance assumption and the normality of data set distribution was assessed using the Levene’s test (*P* < 0.05) (Stanton and Slinker, 1990). For normally distributed data (time course and VIGS gene expression data), comparative analysis was conducted ANOVA incorporating Tukey’s significant difference test at the 0.05% level of significance. For non-normally distributed data (disease scoring data from the VIGS experiment and spore data from the detached leaf experiment), the significance of differences between treatments was assessed using the Kruskal–Wallis test with Dunn’s post-test (Dunn, 1964) in GraphPad Prism (version 5.03 for Windows; GraphPad Software, San Diego, CA, United States).

## Results

### Wheat *TaLRRK-6D* Is a *Poaceae*-Specific LRR-RLK

A previous barley microarray study highlighted that a LRR gene on chromosome 6H, *HvLRRK-6H*, was activated in response to *F. culmorum* in seedling tissue at 48 h post-inoculation (Ali et al., unpubl. data). Bioinformatics analyses showed that the most homologous gene in wheat was on the long arm of chromosome 6D of the cv. Chinese Spring genome and hence it was named *TaLRRK-6D.* We cloned and sequenced the *TaLRRK-6D* mRNA sequence from the FHB resistant wheat cv. CM82936 and the FHB susceptible wheat cv. Remus (see Supplementary Table S3 for gene IDs). The ORFs from the three wheat cultivars (cvs. Chinese Spring, Remus and CM82036) shared >97% nucleotide identity. However, at the protein level, the homology was lower, particularly for the cv. Chinese Spring (Supplementary Table S5 and Supplementary Figure S1). The deduced TaLRRK-6D protein from cv. Chinese Spring shared 73% identity with those from cvs. CM82036 and Remus, and 94% homology with a deduced protein encoded by the unpublished stripe rust-responsive gene ID GU84176 (wheat genotype not specified). Deduced protein homeologues on chromosomes 6A and 6B shared 13-53% identity with the chromosome 6D homeologs from cvs. Chinese Spring, CM82036 and Remus, the 6B variant being particularly divergent at the *N* terminal region (∼13% identity to the cvs. Chinese Spring, CM82036 and Remus chromosome 6D variants) (Supplementary Table S5 and Supplementary Figure S1). Another group of homologous genes were detected on cv. Chinese Spring chromosome 2AL (*TaLRRK-2A:CS*), 2BL (*TaLRRK-2B:CS*), and 2DL (*TaLRRK-2D:CS*), which shared 40.4-58.9% identity with *TaLRRK-6D* variants from the three wheat genotypes (Supplementary Table S5).

**FIGURE 1:**
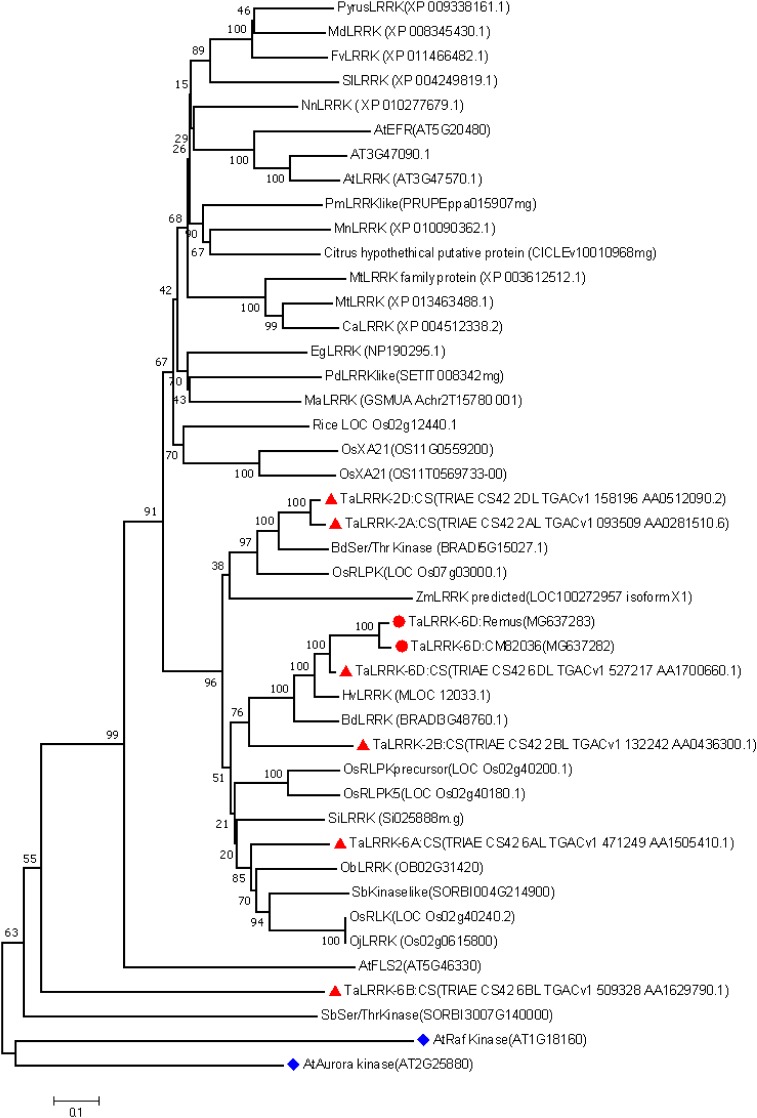
Phylogenetic analysis of the relationship between wheat TaLRRK-6D and homologs from across the plant kingdom. Plant homologs of TaLRRK-6D were identified by BLASTp using EnsemblPlants database.^3^ The cv. Chinese Spring (CS) chromosome 6A, 6B, 6D, 2A, 2B, and 2D TaLRRK variants are highlighted by red triangles; the *TaLRRK-6D* variants from cvs. CM82036 and Remus are highlighted by red circles; outgroups are highlighted by blue squares. Sequences were MAFFT-aligned (Katoh et al., 2002) using Blosum62 matrix (Eddy, 2004) and mean pairwise identity values were used to generate a phylogenetic tree using Jukes Cantor genetic distant model and the Neighbor joining tree building method within the Geneious Tree builder (Kearse et al., 2012) with bootstrapping (1000 replications). Species abbreviations: Sb, *Sorghum bicolor*; Os, *Oryza sativa*; Ob, *Oryza brachyantha*; Oj, *Oryza japonica*; Bd, *Brachypodium distachyon*; At, *Arabidopsis thaliana*; Hv, *Hordeum vulgare*; Pc, *Pyrus calleryana*; Ta, *Triticum aestivum*; Eg, *Elaeis guineensis*; Fg, *Fragaria vesca*; Ma, *Musa acuminate*; Md, *Malus domestica*; Mn, *Morus notabilis*; Ca, *Cicer arietinum*; Cc, *Citrus clementina*; Pm, *Prunus mume*; Pd, *Phoenix dactylifera*; Mt, *Medicago truncatula*; Nn, *Nalumbo nucifera*; Zm, *Zea mays*, and Si, *Setaria italica*.

Phylogenetic analysis shows the TaLRRK-6D protein from cvs. CM82036, Remus and Chinese Spring clusters with proteins from other *Pooaceae* plants (**Figure 1**). All homologs from other non-*Poaceae* families clustered with the 6B homeolog from cv. Chinese Spring, which is a shorter protein that is completely devoid of the signature LRR containing ectodomain of LRR-RLKs (Supplementary Figure S2). The deduced TaLRRK-6D, TaLRRK-6A, TaLRRK-2B, and TaLRRK-2D proteins from CS and TaLRRK-6D from cvs. CM82036 and Remus contains leucine rich repeats in its’ ectodomain (that are crucial for protein–protein interactions; (Kobe and Deisenhofer, 1994), a transmembrane domain and a signal peptide, all signatures of membrane-localized RLK proteins (Shiu and Bleecker, 2001; Rameneni et al., 2015) (Supplementary Figure S2). The protein also encoded a putative concanavalin A-like lectin/glucanase domain (IPR013320) sandwiched within the transmembrane domain (Supplementary Figure S2). Phylogenetic analysis, protein domain and motif analysis firmly place TaLRRK-6D in the LRR-RLK receptor protein subfamily XII (Liu et al., 2017), the closest rice and *Arabidopsis* homologs being LOC Os02g12440 and AT3G47090.1, respectively.

### *TaLRRK-6D* Expression Is Part of the Early Wheat Response to FHB Disease and DON

**FIGURE 2:**
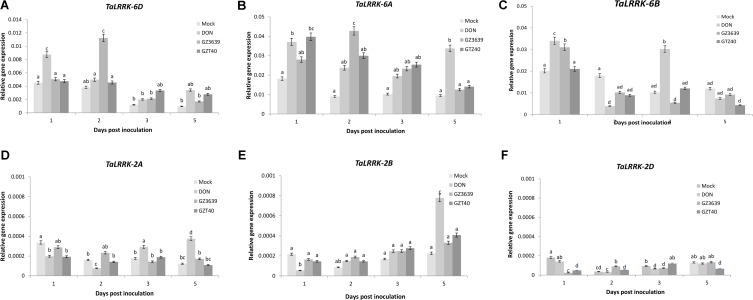
Accumulation of transcript from *TaLRRK-6D* and its homeologs/homologs on chromosomes 2A, 2B, 2D, 6A, and 6B in wheat heads in response to *Fusarium graminearum* or its toxigenic virulence factor deoxynivalenol (DON). Transcripts: **(A)** *TaLRRK-6D*, **(B)** *TaLRRK-6A*, **(C)** *TaLRRK-6B*, **(D)** *TaLRRK-2A* **(E)** *TaLRRK-2B*, and **(F)** *TaLRRK-2D.* Spikes of wheat heads (Fusarium head blight resistant cv. CM82036) were treated with either mock (Tween20), DON or conidia of wild type DON-producing *F. graminearum* strain GZ3639 or its DON-minus mutant derivative GZT40. Treated spikelets were harvested at different time points post-treatment as indicated in the figure legend. Expression of *TaLRRK-6D* and its variants was measured relative to that of the housekeeping genes α*-tubulin* and *GAPDH2.* Results represent mean data obtained from 2 trials for FHB and DON (and in each biological replicate, RNA was extracted from a bulk of four heads per treatment per time point and qRT-PCR was conducted twice per bulk RNA sample). Bars indicate SEM. Treatments with the same letter are not significantly different (*P >* 0.05).

**FIGURE 3:**
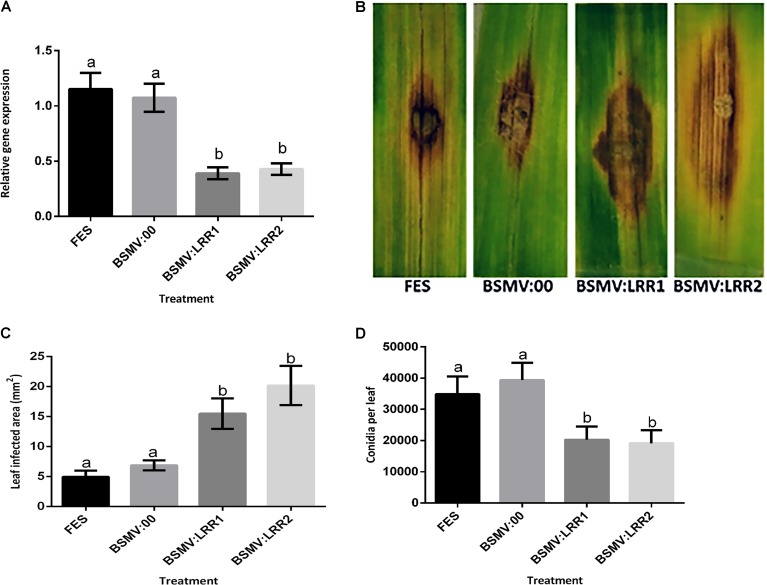
Effect of virus induced gene silencing (VIGS) of *HvLRRK-6H* on the susceptibility of detached barley (cv. Akashinriki) leaves to *Fusarium culmorum.* Plants were treated with either FES (the VIGS buffer), BSMV:00 (empty vector) or BSMV:LRR1 or BSMV:LRR2 (constructs targeting *HvLRRK-6H*). VIGS treatments were applied to the second leaf and the third leaf was detached and treated with a droplet of *F. culmorum* conidia. **(A)** Gene silencing of *HvLRRK-6H* in barley leaves was quantified by real-time PCR analysis using reference genes barley actin (*HvActin)* and α-tubulin (*Hvα-tubulin)* and the 2ˆ-ΔCt method (Livak and Schmittgen, 2001). **(B)** Symptoms of leaf necrosis 4 days post-inoculation of spores on *HvLRRK-6H* silenced Akashinriki lines. **(C)** Quantification of area of infection using image J software in pixel count and converted to area of (2000 pixel = 0.1 cm^2^). **(D)** Macroconidia production by *Fusarium* on the inoculated leaf segments. Results represent mean data obtained from 3 trials (RNA was extracted from individual leaves and then equivalent amounts were bulked from the two leafs from each plate, resulting in 10 bulked RNA samples per treatment combination per trial). Bars in graphs indicate SEM. Treatments with the same letter are not significantly different (*P >* 0.05).

**FIGURE 4:**
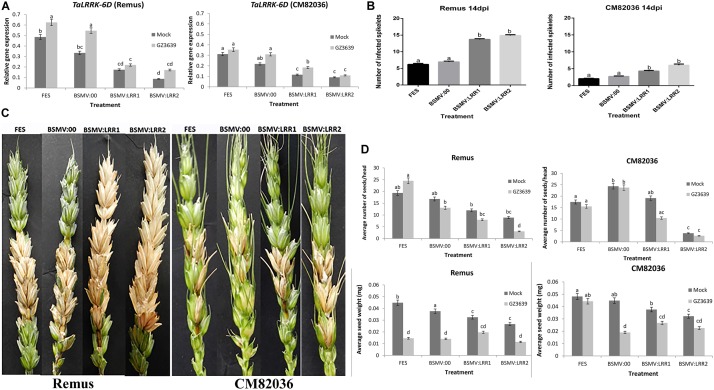
Virus-induced gene silencing (VIGS) of *TaLRRK-6D* in wheat spikelets. Plants of wheat cv. CM82036 and Remus were subjected to VIGS using barley stripe mosaic virus (BSMV) constructs. Plants were treated with either FES (the VIGS buffer), BSMV:00 (empty vector) or BSMV:LRR1 or BSMV:LRR2 (constructs targeting *TaLRRK-6D*). Flag leaves were treated with virus prior to emergence of the first head, and at mid anthesis (growth stage Zadoks 65) the two central florets of the spikelet were inoculated with either mock (Tween20) or conidia of wild type DON-producing *F. graminearum* strain GZ3639. The third spikelet above the treated spikelets was collected for gene expression studies. **(A)** *TaLRRK-6D* gene silencing in wheat spikelets was quantified by real-time PCR analysis using reference genes *α-tubulin* and *GAPDH2* and the 2ˆ-ΔCt method (Livak and Schmittgen, 2001). Disease symptoms were scored at 14 days post-*Fusarium* treatment. **(B)** Images displaying typical disease symptoms. **(C)** Quantification of the number of diseased spikelet per head to assess the disease progression in both CM82036 and Remus cultivars. **(D)** Yield analysis expressed as average number of seeds per head and average seed weight. Disease and yield results represent mean data obtained from 60 heads (20 heads per treatment combination in each of three trials) while gene expression represents 40 heads (RNA from five bulks of four heads per treatment combination from two of the three trials). Bars in graphs indicate SEM. Treatments with the same letter are not significantly different (*P >* 0.05).

Gene variant-specific qRT-PCR assays were developed and used to determine whether *TaLRRK-6D* or its homeologs/homologs on chromosome 6A, 6B, 2A, 2B, and 2D were expressed in heads of the FHB resistant cv. CM82036 in response to *F. graminearum. TaLRRK-6D* expression was induced at 2 dpi, with the effect of the fungus decreasing thereafter (**Figure 2A**; note that in wheat cv. Remus, it was activated earlier at 1 dpi as determined via subsequent gene silencing experiments illustrated in **Figures 3** and **4**). The 6A and 6B homeologs were also up-regulated in cv. CM82036 in response to FHB (**Figures 2B,C**). But for the 6B homeolog, the pathogen induction peaked earlier (1 dpi) as compared to the 6A and 6D homeologs. Although the basal expression of *Ta-LRRK-6D* was lower as compared to either the 6A or 6B homeologs, the responsiveness to the pathogen was higher than that of 6B (peaks of 3.10 and 1.01-fold induction at 1 and 2 dpi, respectively) and lower than that observed for the 6A homeolog (peaking at 4.3-fold induction at 2 dpi) (**Figure 2**). The response of the 6A homeolog to FHB was more sustained than that of the chromosome 6B and 6D variants. The FHB-responsiveness of the *TaLRRK-2A, TaLRRK-2B*, and *TaLRRK-2D* homologs was also analyzed. None of these homeologs were responsive to the pathogen from 1 to 5 dpi (**Figures 2D–F**).

*Fusarium graminearum* is a hemibiotroph (Sutton, 1982; Kazan and Lyons, 2014). In the initial stages of infection, it behaves as a biotroph, feeding off living host tissue, and later (approx. 72 h post-infection), it switches to a necrotrophic lifestyle, feeding off dead host tissue (Oliver and Ipcho, 2004; Król et al., 2015). DON has been shown to facilitate the necrotrophic phase of disease (Bai et al., 2002; Maier et al., 2006; Cuzick et al., 2008) and resistance to the phytotoxic effects of DON is an innate component of FHB resistance (Miller et al., 1985; Mesterházy, 2002; Gunupuru et al., 2017). Thus, in the temporal gene expression experiment, we also analyzed the effect of DON on *TaLRRK-6D* gene expression and found that the gene was induced as an early response to the toxin at 1 day post-treatment, expression returning to levels comparable to the mock treatment by 2 days (**Figure 2A**). In order to confirm that endogenous fungal DON levels produced during wheat infection could induce *TaLRRK-6D*, we included an additional fungal treatment in the time course experiment – i.e., we treated wheat heads with GZT40 which is a non-DON-producing mutant of wild type strain GZ3639 (Proctor et al., 1995). Unlike wild type strain GZ3639, mutant GZT40 did not significantly induce *TaLRRK-6D* expression at 1 dpi or at any other time point assessed (**Figure 2A**). Previously, QPCR was used to analyze fungal actin levels as an indicator of fungal biomass in the same RNA extracts and levels for the mutant and wild type was not significantly different up to 5 dpi (Perochon et al., 2015). Thus, the lack of *TaLRRK-6D* induction by the mutant was not reflective of biomass levels. Hence, we conclude that DON production by *F. graminearum* facilitates the early induction of *TaLRRK-6D* as part of the wheat defense response against FHB disease. *TaLRRK-6B* was similar to the chromosome 6D gene in that both were induced by DON and wild type-*F. graminearum*, but not by the non-DON producing mutant strain. However, unlike the 6D variant, induction of *TaLRRK-6B* by DON was biphasic, occurring at both 1 and 3 dpi (**Figure 2C**). In contrast to *TaLRRK-6B* and *TaLRRK-6D*, the *TaLRRK-6A* homeolog was induced by wild type and DON-minus mutant fungus (and by DON) (**Figure 2B**). The 6A variant was significantly induced at both day one and day five by DON, but the biphasic nature was not as clear as for the 6B gene (expression levels in response to DON being statistically similar at all 4 days’ *P >* 0.05). The expression of the 2A, 2B, and 2D homeologs were very low relative to that of 6A, 6B, and 6D variants: none were induced by *F. graminearum* and induction of the 2A and 2B homeologs by DON did not occur until 5 dpi (**Figures 2D–F**).

### Barley *HvLRRK-6H* Contributes to Leaf Resistance to *F. culmorum*

Virus-Induced Gene Silencing was used to determine if silencing the barley *HvLRRK-6H* altered the hosts’ ability to resist *Fusarium* infection. We designed two independent non-overlapping, gene-specific VIGS constructs (BSMV:LRR1 and BSMV:LRR2; Supplementary Table S3) that can target both the barley *HvLRRK-6H* and wheat *TaLRRK-6D* for silencing. We used the detached leaf assay to assess the effect of *HvLRRK-6H* silencing on the response of cv. Akashinriki to *F. culmorum*. Gene-specific qRT-PCR of leaves at 7 days post-pathogen inoculation confirmed that VIGS worked efficiently. *Fusarium* treatment induced *HvLRRK-6H*, but in gene-silenced plants (BSMV:LRR1 and BSMV:LRR2) the expression of *HvLRRK-6H* was significantly reduced by 64%, as compared to the effect of *F. culmorum* on plants treated with the mock virus (BSMV:00) (*P* ≤ 0.05) (**Figure 3A**). At a phenotypic level, treatment with either BSMV:LRR1 or BSMV:LRR2 led to respective 2.6 and 3.2-fold increases in disease lesion size by 4 dpi, relative to BSMV:00-treated plants (**Figures 3B,C**). These results suggest that a functional *HvLRRK-6H* is important for barley resistance to *Fusarium* infection. Thus, we concluded that *HvLRRK-6H* contributes to barley resistance to *F. culmorum.* As stated earlier, *HvLRRK-6H* was originally identified as being up-regulated in the uzu derivative of cv. Akashinriki, as compared to the parental line, in response to *Fusarium.* A VIGS study in uzu (conducted concurrently with the VIGS study in cv. Akashinriki) validated that *HvLRRK-6H* also contributed to leaf resistance in this kinase mutant barley derivative (Supplementary Figure S3). Notably, there was no evidence that uzu leaves were more resistant to *Fusarium* than the wild type parent (comparing **Figure 3** and Supplementary Figure S3), suggesting that the *Fusarium* resistance of uzu is not manifested in the leaves.

Completion of the disease cycle requires the production of conidia that serve as inoculum for the infection of new plants Perochon et al. (2015) recently found that the *Pooideae*–specific orphan gene *TaFROG* inhibited lesion development and the number of spores produced by *Fusarium* on wheat leaves. We also assessed the effect of *HvLRRK-6H* silencing on the quantity of spores produced by *F. culmorum* on barley leaves and found that BSMV:LRR1 and BSMV:LRR2–treated sections both contained two-fold less rather than more conidia as compared to BSMV:00 treated plants (*P* < 0.05) (**Figure 3D**). Thus we conclude that either the increased lesion size due to gene silencing did not positively affect sporulation at the time point analyzed (i.e., there was no positive association between disease lesion size and conidia as seen for other resistance genes, e.g., Perochon et al., 2015) and/or that the controls contained more ungerminated conidia at this time, relative to the gene-silenced leaves.

### Wheat *TaLRRK-6D* Contributes to FHB Disease

We used VIGS to determine if *TaLRRK-6D* contributes to wheat defense against FHB in heads of both a disease resistant and susceptible genotype (cvs. CM82036 and Remus, respectively). The VIGS constructs BSMV:LRR1 and BSMV:LRR2 used above for barley were also used for wheat as they can also target *TaLRRK-6D* for silencing (Supplementary Table S3). The constructs specifically targeted the wheat variant on chromosome 6D for silencing (**Figure 4A**), as compared to either the chromosome 6A, 6B, 2A, 2B, or 2D variants (see Supplementary Figure S4). VIGS did not silence the 6A and 6B genes and the expression of the 2A, 2B, and 2D variants was very low, irrespective of treatment. *TaLRRK-6D-*specific qRT-PCR of heads at 1-day post-*Fusarium* treatment validated that, in the absence of gene silencing (FES buffer treatment or empty virus BSMV:00 treatment), the expression of *TaLRRK-6D* was lower in cv. CM82036 than in cv. Remus, and at this time point the gene was significantly upregulated by *Fusarium* in cv. Remus but not in cv. CM82036 (*P* ≤ 0.05). Silencing by either VIGS construct (BSMV:LRR1 or BSMV:LRR2) reduced the transcription of *TaLRRK-6D* in the two wheat genotypes (**Figure 4A**). In non-fungal treated heads, treatment with BSMV:LRR1 or BSMV:LRR2 reduced transcript levels by 40–86% in cvs. CM82036 and Remus, and relative to plants treated with the empty virus (BSMV:00). Effects of VIGS on gene expression in fungal treated tissue reflected the effects observed in mock-treated tissue (reductions of 42–69%, relative to BSMV:00; **Figure 4A**). At the phenotype level, the assessment of heads at 14 days post pathogen treatment showed that, in both wheat genotypes, BSMV:LRR1 and BSMV:LRR2 plants were 2.7-fold more diseased than BSMV:00 plants (**Figures 4B,C**; Supplementary Figure S5 shows that similar results were obtained at 7 dpi). By 21 dpi, it should be noted that pink fungal growth was visible on diseased spikelets of plants wherein the *LRR* gene was silenced, often embedded with black sexual spores structures (Supplementary Figure S6).

At harvest, seed numbers and seed dry weight were calculated. The mock virus treatment BSMV itself affected grain development, but to a lesser extent than the gene silencing constructs, particularly BSMV:LRR2 (**Figure 4D**). The most striking results for grain was that gene silencing, relative to BSMV, retarded grain development in healthy non-diseased heads. In non-fungal treated heads of the two cultivars, silencing of *TaLRRK-6D* resulted in a 21–85% reduction in the average number of seeds per head and a 28–69% reduction in grain weight, as compared to BSMV:00 treatment (**Figure 4D**). Thus, we conclude that *TaLRRK-6D* reduces the severity of the disease symptoms caused by FHB and it also positively contributes to grain development. FHB effects on grain number and weight were usually not significantly exacerbated due to gene silencing (an interesting aside was that the empty virus BSMV negated the FHB resistance in cv. CM82036 in terms of the effect of disease on seed weight).

### Silencing *TaLRRK-6D* Down Regulates SA Signaling Genes

**FIGURE 5:**
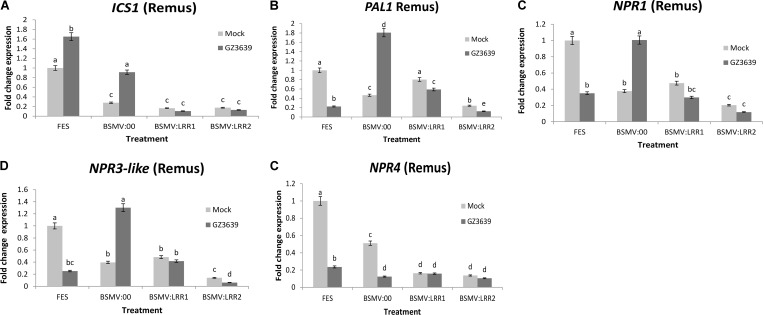
Effect of *TaLRRK-6D* silencing on the expression pattern of SA signaling genes in wheat heads challenged with *Fusarium graminearum*. The relative expression of SA signaling pathway genes were analyzed in VIGS *TaLRRK-6D* silenced spikelets samples of wheat cv. Remus 1 dpi of *Fusarium*. SA signaling transcripts: **(A)** *ICS1*, **(B)** *PAL1*, **(C)** *NPR1***, (D)** *NPR3-like*, and **(E)** *NPR4* showing independent expression with *Fusarium* treatment and effects of *TaLRRK-6D* silencing on its expression. The relative expression was calculated using an efficiency corrected model using the formula [(Etarget)^ΔCt target (control-sample)^/ (E_housekeeping_)^ΔCt housekeeping (control-sample)^] (Souaze et al., 1996). Thereafter, the values are expressed relative to the fold change for the FES mock treatment. Results represent mean data obtained from 40 heads (two trials and in each RNA was obtained from five bulks of four heads per treatment combination). Bars in graphs indicate SEM. Treatments with the same letter are not significantly different (*P >* 0.05).

The involvement of SA signaling in crop defense against the biotrophic phase of FHB disease has been demonstrated via studies on the effect of exogenous SA on key signaling genes (Makandar et al., 2012; Sorahinobar et al., 2016). As shown above, gene expression studies validated that *TaLRRK-6D* is systemically activated by *F. graminearum* during this phase, as early as 1 dpi in cv. Remus (**Figure 4**) and that VIGS of this gene enhanced FHB severity; resulting in very high disease levels in cv. Remus (**Figure 4**). We thus postulated that *TaLRRK-6D* might be an upstream and important component of SA defense against FHB. To test this hypothesis, we analyzed the effect of VIGS of *TaLRRK-6D* in cv. Remus on the expression of genes involved in SA accumulation, signaling and perception (using qRT-PCR studies of the RNA from tissue harvested 1-day post *F. graminearum* treatment). *ICS1* and *PAL* are key SA accumulation genes as shown in *Arabidopsis* (Dempsey et al., 2011); *PAL1*, but not *ICS1*, was activated during wheat defense against *Fusarium* (Ding et al., 2011; Makandar et al., 2012; Sorahinobar et al., 2016). The silencing of *TaLRRK-6D* resulted in the down-regulation of both *ICS1* and *PAL* in both mock and *F. graminearum-*treated tissue (**Figures 5A,B**). It is noteworthy that *ICS1* was activated by *Fusarium* in this wheat genotype. The non-induction of *PAL1* as compared to *ICS1* upon *Fusarium* infection may be a time factor as the genes targeted by the qRT-PCR primers have been shown to be activated in wheat in response to FHB disease at 30 and 48 or 50 h post-inoculation (Kugler et al., 2013; Xiao et al., 2013) (see Supplemental Results). The key SA regulator gene *NPR1* and negative regulators *NPR3-like and NPR4* are all induced by either SA (Liu et al., 2005) or by the SA analog 2,6-dichloroisonicotinic acid (INA) (Zhang et al., 2006) (in *Arabidopsis*) and the silencing of *TaLRRK-6D* in wheat cv. Remus led to the downregulation of these three genes (**Figures 5C–E**), indicative of reduced SA signaling. At the time point assessed (1 dpi) these genes were repressed rather than induced by *Fusarium* (see FES treatment; an interesting aside was that the results for the BSMV:00 mock viral treatment demonstrates that both *NPR1* and *NPR3-like* were down-regulated by the virus used for VIGS but induced by the combination of virus and *Fusarium*). Like the *PAL1* results, findings must be interpreted with caution as the time point (1 dpi) may be too early for *Fusarium* induction of these genes in this genotype.

## Discussion

Herein we have identified and characterized a wheat *LRR* gene and deduced that it and its’ barley homolog both contribute to cereal disease resistance. The results for *TaLRRK-6D* concur with previous studies, which suggest that LRR genes are induced as part of the early cereal response to FHB disease. A LRR-RLK gene and NBS-LRR genes were induced as part of the early response to FHB disease in wheat (Guo et al., 2006; Subramaniam et al., 2009; Ravensdale et al., 2014; Kosaka et al., 2015) and barley (Huang et al., 2016). *TaLRRK-6D* induction by *F. graminearum* was in direct response to the fungal virulence factor DON. We thus deduce that *TaLRRK-6D* is a signaling molecule involved in the wheat response to DON. It is possible that the induction might be due to the immediate downstream defense responses that are activated in response to DON production by the fungus. It is known that DON has the ability to trigger the production of reactive oxygen species (ROS) and defense gene induction in wheat (Desmond et al., 2006). Two LRR-RLKs (*TaRLK1* and *TaRLK2)* from wheat have been linked to altered ROS homeostasis in the defense response to *Blumeria graminis* f.sp. *tritici* (*Bgt*) infection. The expression of these *TaRLK* genes was induced upon hydrogen peroxide application and in wheat overexpressing *TaRLK* there was increased hydrogen peroxide accumulation at the *Bgt* penetration sites (Chen et al., 2016). The enhanced ROS production during *F. culmorum* infection of uzu as compared to parent barley lines (Ali et al., 2014) suggest that the kinase activity of the LRR-RLK BRI1 might not be essential for defense responses, but this requires further validation.

ROS and defense gene induction are downstream components of PTI which is activated as a result of PAMPs interacting with RLKs such as TaLRRK-6D (Zipfel, 2014; Silva Couto and Zipfel, 2016). The *Fusarium* induction of *TaLRRK-6D* (and the 6A and 6B homeologs) peaks during the biotrophic phase of FHB disease, i.e., within the first 72 h post-inoculation. ROS accumulation would be a valuable counter-attack against biotrophism. It remains to be determined whether *TaLRRK-6D* plays a role in ROS accumulation or cell death signaling. Similarly, SA is a downstream component of PTI (Newman et al., 2013) and the reduced transcription of SA biosynthesis, regulator and receptor genes as a result of *TALRRK-6D* silencing, together with the effects of exogenous SA in the early wheat defense against FHB (Makandar et al., 2012), further validate the importance of the RLK as an important component of early cereal defense against *F. graminearum.* VIGS of *TALRRK-6D* reduced the amount of *ICS1, PAL1, NPR1 NPR3-like*, and *NPR4* trancript in wheat heads at 1 dpi. VIGS of *ICS1* also negated the *Fusarium* induction of the *ICS1* gene at 1 dpi. Reduced *ICS1* transcript is indicative of reduced SA acummulation in *TALRRK-6D-*silenced plants; defective *ICS1* in *Arabidopsis* led to a 90% reduction in SA accumulation in wild-type plants upon pathogen challenge (Dewdney et al., 2000). Reduced SA levels leading to reduced signaling upon *TALRRK-6D* silencing is supported by reduced basal *PAL1, NPR1, NPR3-like*, and *NPR4* transcript levels, but the time assessed (1 dpi) was likely too early to analyze any *Fusarium* induction of these genes; indeed, it is interesting to note that they were repressed by the pathogen at this time. The negative effect of VIGS on the basal expression of all SA pathway genes analyzed (and on the early *Fusarium* induction of *ICS1*) leads us to hypothesize that TaLRRK-6D is upstream of SA signaling and that silencing of this gene downregulates or attenuates SA signaling in wheat.

The sequence diversity between TaLRRK-6D from different species, genotypes and between this protein and its homeologs is not unexpected. A high level of intrachromosomal segmental (SD) and tandem (TD) duplication among wheat TaLRRKs from chromosomes 6 and 2 has been reported (Shumayla et al., 2016). Similar LRR-RLKs sequence diversity has been reported for homologs of a maize wall-associated receptor-like kinase (ZmWAK-RLK1); the extracellular domain of ZmWAK-RLK1 is highly diverse between different maize genotypes (Hurni et al., 2015). Such diversity in domain composition has also been reported for LRR-RLKs from rice (Sun and Wang, 2011) and brassica (Rameneni et al., 2015). The variation in domain composition found in LRR-RLKs may support the deviation in signal perception and diverse biological roles. There was a clear distinction between the three chromosome 6 *TaLRRK* homeologs with respect to their temporal and DON-dependent responsiveness to *F. graminearum.* Unlike 6D, the chromosome 6A homeolog also induced by the DON-minus mutant of the fungus (therefore it was not specific to DON), and the 6B homeolog was clearly induced by DON in a biphasic manner (with a less defined biphasic pattern for the 6A homeolog). The biphasic induction of the 6B homeolog is reminiscent of the biphasic oxidative burst that occurs in many incompatible plant-pathogen interactions, whereby an initial localized burst of ROS is linked to a secondary systemic phase of ROS production (Ren et al., 2006; Torres et al., 2006; Liu et al., 2007; Zurbriggen et al., 2010). Sub-functionalisation of homeologous wheat genes is a relatively new area of study: Powell et al. (2017) recently showed that homeolog expression bias underpins a large proportion of the wheat transcriptome. The temporal and stimulus-specific differences in the FHB induction of these homeologous LRRK genes suggests that the study of their role in disease resistance provides an interesting model to improve our understanding the subtleties of how polyploidy has contributed to the sophistication of wheat defense responses.

When comparing the *TaLRRK-6D* expression in Remus (mock and *Fusarium*) versus CM82036, we found that the expression of *TaLRRK-6D* was always higher. The VIGS analysis indicated that wheat *TaLRRK-6D* and its barley homolog *HvLRRK-6H* positively contributes to resistance to *F. graminearum.* The VIGS study in wheat also confirmed that *TaLRRK-6D* contributed to defense in both FHB-resistant and susceptible wheat genotypes, with gene silencing enhancing visual disease symptom development. Whether this is true for the *Fusarium-*responsive wheat A and B homeologs remains to be determined. *HvLRRK-6H* is the second LRR shown to contribute to barley resistance to FHB disease. *HvLRRK-6H* was originally highlighted as being overexpressed in uzu barley lines in which the gene encoding the LRR receptor kinase brassinosteroid-insentitive 1 (BRI1) is mutated (Ali et al., unpubl. data). Ali et al. (2014) demonstrated that BRI1 contributes to barley resistance to *Fusarium* in both seedling and flowering tissue. VIGS of another barley LRR receptor kinase responsive to both powdery mildew (*Blumeria graminis* f. sp. *hordei*) and stem rust (*Puccinia graminis* f. sp. *tritici*) resulted in reduced defense genes expression, suggesting a tentative PRR role against these fungal pathogens (Parrott et al., 2016). *TaLRRK-6D* and *HvLRRK-6H* add a cereal gene to the list of LRR-RLK sub-family LRR XII genes with proven roles in defense against pathogens; this sub-family also includes *Arabidopsis* FLS2, *Arabidopsis* EF-Tu receptor and rice Xa21 (Shiu et al., 2004; Schoonbeek et al., 2015; Schwessinger et al., 2015; Thomas et al., 2017).

The reduced grain weight due to the silencing of *TaLRRK-6D* suggests that it may have role to play in wheat yield components, as previously reported for a rice LRR-RLK. The overexpression of rice LRR-RLK *OsLRK1* gene led to a 27% increase in total grain yield per plant (Zha et al., 2009). Recently, overexpression of the wheat LRR-RLK *TaBRI1* in *Arabidopsis* was found to induce early flowering, increased silique size and increased seed yield (Singh et al., 2016). *TaLRRK-6D* is not the first FHB resistance gene associated with grain development; a wheat ABC transporter (Walter et al., 2015) and cytochrome P450 (Gunupuru et al., unpubl. data) were found to contribute to grain formation. And a genome-wide gene expression profiling of the wheat (in a FHB susceptible cultivar) found that the FHB responsive transcriptome was enriched in genes involved in grain development (Chetouhi et al., 2016). A global expression analysis of rice RLK indicated that they are important players during embryo and endosperm development (Gao and Xue, 2012).

To conclude, this study highlighted the contribution of specific wheat and barley leucine rich repeat receptor like kinase homologs to *Fusarium* resistance. These should be further investigated as potential candidate genes for both GM and breeding programs that aim to enhance *Fusarium* resistance in cereals. Based on the current wheat genome sequence, these genes do not collocate with known FHB resistance genetic loci on chromosome 6D. Ongoing studies will determine if variation in the *TaLRRK-6D* gene/gene promoter or its homeologs contribute to quantitative resistance to FHB. The gene expression studies suggest that the 6A and 6B variants are also important components of the FHB response in wheat and these also merit further study to determine if they contribute to disease resistance. Ultimately, the determination of the interacting partners and potential ligand(s) of TaLRRK proteins will help us better understand the cellular mechanisms underlying FHB resistance and the durability of such defense strategies.

## Author Contributions

GT and FD planned and designed the research. GT, LG, JH, and AK performed the experiments. GT, EM, and FD analyzed the data. GT and FD wrote the manuscript.

## Conflict of Interest Statement

The authors have a patent pending related to this material.

## Footnotes

1 http://www.jic.ac.uk/germplasm/

2 http://plants.ensembl.org/*Triticum_aestivum*/Tools/Blast?db=core

3 http://plants.ensembl.org

4 https://www.geneious.com

5 http://smart.embl-heidelberg.de

6 http://prosite.expasy.org/

7 http://hamap.expasy.org/

8 http://www.bioinf.manchester.ac.uk/

9 http://pfam.xfam.org/

10 http://supfam.org/SUPERFAMILY/

11 https://www.ebi.ac.uk/interpro/

12 http://labtools.ipk-gatersleben.de/index.html

13 http://plants.ensembl.org/*Triticum_aestivum*

14 http://www.wheat-expression.com/

15 https://www.ebi.ac.uk/gxa/about.html

16 https://www.ncbi.nlm.nih.gov/sra
