# Supplementary material for: A Pathogen-Responsive Leucine Rich Receptor Like Kinase Contributes to Fusarium Resistance in Cereals
Source: Front Plant Sci. 2018 Jun 26;9:867. doi: 10.3389/fpls.2018.00867 (PMC6029142; doi:10.3389/fpls.2018.00867)
Supplement: Supplementary file 1 [file Table_1.DOCX]

**Supplementary Table S1.** **List of the nullisomic-tetrasomic lines used in the study.**

| **Index Number** | **Description** | **Source** |
| --- | --- | --- |
| WPGS1128 | *Triticum aestivum* Cv. Chinese Spring, Nulli2A Tetra2B | Germplasm Research Unit, JIC, NR47UH, UK |
| WPGS1139 | *Triticum aestivum* Cv. Chinese Spring, Nulli2A Tetra2D | Germplasm Research Unit, JIC, NR47UH, UK |
| WPGS1148 | *Triticum aestivum* Cv. Chinese Spring, Nulli2B Tetra2A | Germplasm Research Unit, JIC, NR47UH, UK |
| WPGS5053 | *Triticum aestivum* Cv. Chinese Spring, Nulli2D Tetra2A | Germplasm Research Unit, JIC, NR47UH, UK |
| WPGS1284 | *Triticum aestivum* Cv. Chinese Spring, Nulli6D Tetra6A | Germplasm Research Unit, JIC, NR47UH, UK |
| WGRC13-15 | *Triticum aestivum Cv.* Chinese Spring, Nulli6A Tetra6D | Wheat Genetic Resource Centre, KSU, USA |
| WGRC13-15 | *Triticum aestivum Cv.* Chinese Spring, Nulli6B Tetra6D | Wheat Genetic Resource Centre, KSU, USA |
