# Supplementary material for: A Pathogen-Responsive Leucine Rich Receptor Like Kinase Contributes to Fusarium Resistance in Cereals
Source: Front Plant Sci. 2018 Jun 26;9:867. doi: 10.3389/fpls.2018.00867 (PMC6029142; doi:10.3389/fpls.2018.00867)
Supplement: Supplementary file 2 [file Table_2.DOCX]

**Supplementary Table S2.** **List of primers used in TaLRRK-6D cloning and VIGS analysis.**

| **Primer name** | **5’-3’ sequence** | **Description** | | |
| --- | --- | --- | --- | --- |
| Ta6D515MF | *CTGGTGGTTCTTGACTTGAAA* | *TaLRRK-6D* cloning primers | |  |
| Ta6D1815MR | *GCTGACATGTTGAAGACTTACA* |  |  |  |
| Ta6DUTRF | *AGCTGCCGTTTTGTGCGCTC* |  |  |  |
| Ta6DUTRR | *GTCCTGCTACTTCCCTTCAAAAAC* |  |  |  |
| TaLRRK6DF | *ATGTCTGACCAATCCGTGAAACTC* |  |  |  |
| TaLRRK6DRTra | *AAC TGA AGG ATT TAT CTT TGC CT* |  | |  |
| TaLRRK6DRNcbi | *CTAGTGATTGTTGTAAGTTT* |  |  |  |
| CM/R6D5RaR | *GAGTTTATTCCTAGAAAGGTCTAAATTC* | RACE primers | |  |
| CM6D5RaR | *GTA AAG CGA ACC TAT TTT CGC C* |  |  |  |
| CM/R 6DR’R | *CAT TTG ACC TGA GAG CCT ATT GCT* |  |  |  |
| CM/R 6D3RF | *ATCCAAATGGACCATAATAACT* |  |  |  |
| CM/R 6D3’R’F | *GGAAACTTAAAAGACTCTCAGCCA* |  |  |  |
| Xa21-VIGS1F | *CGATTAATTAATTCGGCATTGCAAGGTTT* | VIGS LRR1 construct primers | |  |
| Xa21-VIGS1R | *CGACCCGGGGGAAAGTTGCTGCACACG* |  |  |  |
| Xa21-VIGS2F | *CGATTAATTAATGCATAACGACAGCGAAAAC* | VIGS LRR2 construct primer | |  |
| Xa21-VIGS2R | *CGACCCGGGTCACACCAATCGAACTCGTC* |  |  |  |
| 2ALRRF | *TTGCACTGCTGACTTGCTTT* | Homeolog-specific real-time primers | |  |
| 2ALRRR | *TGACTGCCACTTCCGTTTTG* |  |  |  |
| 6DLRRF | *CCCTAAACTTGAACTTCTACATCT* |  |  |  |
| 6DLRRR | *TACAGATATGTTAGCTCAGTAAGG* |  |  |  |
| 2BLRRF | *AGTCAATCACGAGAACAGTAGAG* |  |  |  |
| 2BLRRR | *TGGAGGACAAGTTGAGGACC* |  |  |  |
| 2DLRRF | *CCTTGAGTACTTAAACCTTGGGC* |  |  |  |
| 2DLRRR | *AGACTTGTAAGGTGAGTGGACA* |  |  |  |
| 6ALRRKF1 | *AAACTCACTCTCACTGGGTTTC* |  |  |  |
| 6ALRRKR1 | *CCGGTGAAGCGGTTTGAG* |  |  |  |
| 6BLRRKF1 | *GACCAGCAAGGTGTACCG* |  |  |  |
| 6BLRRKR1 | *CACTCGAACACCAGCAGC* |  |  |  |
| TaLRRK6DqRTF | *CCATTCTCGGGAAACTTAAAAG* |  |  |  |
| TaLRRK6DqRTR | *AAGTCTGTTAGATGAAAGATTC* |  |  |  |
| Taα-tubulinF | *ATCTCCAACTCCACCAGTGTCG* (Xiang et al., 2011) | Housekeeping primers | |  |
| Taα-tubulinR | *TCATCGCCCTCATCACCGTC* (Xiang et al., 2011) |  |  |  |
| TaGAPDH2F | TCACCACCGACTACATGACC (Perochon et al, 2015) |  |  |  |
| TaGAPDH2R | ACAGCAACCTCCTTCTCACC (Perochon et al, 2015) |  |  |  |
| FgActinF | *ATGGTGTCACTCACGTTGTCC* (Brown et al., 2011) |  | |  |
| FgActinR | *CAGTGGTGGAGAAGGTGTAACC* (Brown et al., 2011) |  | |  |
| Hvα-tubulinF | *GCATGGAGGAGGGAGAGTTC* (Ali et al., 2014) |  | |  |
| Hvα-tubulinR | *CCAGGAGGCAGGCTCTAGTAC* (Ali et al., 2014) |  | |  |
| HvActinF | *CCACGAGACGACCTACAAC* (Ferdous et al., 2015) |  | |  |
| HvActinR | *CACTGAGCACGATGTTTCC* (Ferdous et al., 2015) |  | |  |
| M13FP(17mer) | *GTTTTCCCAGTCACGAC* | Sequencing primers | |  |
| M13RP(17mer) | *CAGGAAACAGCTATGAC* |  |  |  |
| T7 FP | *TAATACGACTCACTATAGGG* |  |  |  |
| SP6 FP | *TATTTAGGTGACACTATAG* |  |  |  |
