# Supplementary material for: A Pathogen-Responsive Leucine Rich Receptor Like Kinase Contributes to Fusarium Resistance in Cereals
Source: Front Plant Sci. 2018 Jun 26;9:867. doi: 10.3389/fpls.2018.00867 (PMC6029142; doi:10.3389/fpls.2018.00867)
Supplement: Supplementary file 3 [file Table_3.DOCX]

**Supplementary Table S3.** **Specificity of the constructs used for VIGS.**

| **Homeologue** | **GENE ID** | **Cereal/genotype** | **Chromosome** | **Construct BSMV:LRR1 (245bp)** | | | **Construct BSMV:LRR2 (162bp)** | | |
| --- | --- | --- | --- | --- | --- | --- | --- | --- | --- |
|  |  |  |  | **Percentage/length (bp) of homology^a^** | **Predicted silencing^b^** | **Silencing based on qRT-PCR^c^** | **Percentage/length (bp) of homology^a^** | **Predicted silencing^b^** | **Silencing based on qRT-PCR^c^** |
| *TaLRRK-2A* | TRIAE_CS42_2AL_TGACv1_093509_AA0281510.6 | Wheat/Chinese Spring | 2A | 5/11 | No | No | 0/0 | No | No |
| *TaLRRK-2B* | TRIAE_CS42_2BL_TGACv1_132242_AA0436300.1 | Wheat/Chinese Spring | 2B | 12/28 | No | No | 16/26 | No | Genotype-specific^e^ |
| *TaLRRK-2D* | TRIAE_CS42_2DL_TGACv1_158196_AA0512090.2 | Wheat/Chinese Spring | 2D | 10/25 | No | Genotype-specific^e^ | 24/39 | No | No |
| *TaLRRK-6A* | TRIAE_CS42_6AL_TGACv1_471249_AA1505410.1 | Wheat/Chinese Spring | 6A | 51/68 | No | No | 37/49 | No | No |
| *TaLRRK-6B* | TRIAE_CS42_6BL_TGACv1_509328_AA1629790.1 | Wheat/Chinese Spring | 6B | 14/25 | No | No | 11/17 | No | No |
| *TaLRRK-6D* | TRIAE_CS42_6DL_TGACv1_527217_AA1700660.1 | Wheat/Chinese Spring | 6D | 88/245 | Yes (41) | Yes | 97/158 | Yes (83) | Yes |
| *TaLRRK-6D* | MG637282 | Wheat/CM82036 | 6D | 100/231 | Yes (100) | Yes | 100/157 | Yes (100) | Yes |
| *TaLRRK-6D* | MG637283 | Wheat/Remus | 6D | 87/231 | Yes (93) | Yes | 90/157 | Yes (92) | Yes |
| *HvLRRK-6H* | MLOC_12033.1 | Barley/Morex | 6H | 94/231 | Yes (98) | Yes | 97/158 | Yes (86) | Yes |

^a^Homology (%) and longest length (bp) of homology of the VIGS fragment to the *LRR-RLK* variant gene sequence in wheat (cvs. Chinese Spring, CM82036 and Remus) or barley (cv. Morex).

**^b^**Predicted silencing of the *LRR-RLK* variant based on both the homology and the on siRNA finder si-fi tool (labtools.ipk-gatersleben.de/index.html). Note, where relevant, the silencing efficiency based on the si-fi tool is given in parenthesis.

**^c^**Silencing based on qRT-PCR with variant-specific primers in wheat (cvs. CM82036 and Remus) or barley (cv. Akashinriki). Note for the 2B variant in wheat, the silencing by BSMV:LRR2 was genotype-specific (silencing in cv. Remus but not in cv. CM82036).

^e^Basal expression levels are very low and thus any silencing effects should be viewed with caution (see Figure S3).
