# Supplementary material for: A Pathogen-Responsive Leucine Rich Receptor Like Kinase Contributes to Fusarium Resistance in Cereals
Source: Front Plant Sci. 2018 Jun 26;9:867. doi: 10.3389/fpls.2018.00867 (PMC6029142; doi:10.3389/fpls.2018.00867)
Supplement: Supplementary file 4 [file Table_4.DOCX]

**Supplementary Table S4.** **List of primers used to amplify SA biosynthesis, regulator and receptor genes via qRT-PCR**

| **Gene** | **Description/Reference** | **Wheat homeologs/variant target ^a^** | **Primer name and sequence^b^** |
| --- | --- | --- | --- |
| *ICS1* | SA biosynthesis in *Arabidopsis* (Dempsey et al., 2011) | TRIAE_CS42_5BL_TGACv1_407642_AA1358550.3; TRIAE_CS42_5DL_TGACv1_433720_AA1420520.2;  TRIAE_CS42_5AL_TGACv1_378731_AA1254400.1; | ICS1F-CGGACGGCCCCGCCGAGGAC  ICS1R- TCGCTCGACGCGTCGAAACG |
| *PAL1* | SA biosynthesis in *Arabidopsis* (Dempsey et al., 2011) | TRIAE_CS42_2AL_TGACv1_093330_AA0277780.1; TRIAE_CS42_2AL_TGACv1_093330_AA0277780.2; TRIAE_CS42_2BS_TGACv1_146234_AA0459760.1; TRIAE_CS42_2DS_TGACv1_178157_AA0592020.1; TRIAE_CS42_1BS_TGACv1_049914_AA0164170.1; TRIAE_CS42_1BS_TGACv1_049914_AA0164170.2 | PAL1F-CGACGAGGTCAAGCGCATGGT  PAL1R-CGGCTGCTCTCCTTGACGCGG |
| *NPR1* | SA perception in *Arabidopsis* (Liu et al., 2005)  SA receptor in *Arabidopsis* (Zhang et al., 2006)  SA receptor in *Arabidopsis* (Liu et al., 2005) | TRIAE_CS42_3AS_TGACv1_211317_AA0688550.1; TRIAE_CS42_3DS_TGACv1_272064_AA0914040.1; TRIAE_CS42_3DS_TGACv1_272064_AA0914040.2 | NPR1F1-AGACAATGGCTTTCCTAACAAA  NPR1R1-GATGTGAAGAACAGTATAACCT |
| *NPR3-like* |  | TRIAE_CS42_3B_TGACv1_221761_AA0749410.1;  TRIAE_CS42_3AL_TGACv1_193833_AA0620480.3; TRIAE_CS42_3DL_TGACv1_249840_AA0857170.1; TRIAE_CS42_3DL_TGACv1_249840_AA0857170.2 | NPR3F-ATGGAGCCGTCGTCGTCCATCA  NPR3R-TCCGCCACGTCGACGTCGGCGT |
| *NPR4* |  | TRIAE_CS42_5DS_TGACv1_457109_AA1482480.4; TRIAE_CS42_5BS_TGACv1_428630_AA1393500.1; TRIAE_CS42_5DS_TGACv1_457109_AA1482480.5; TRIAE_CS42_5DS_TGACv1_457505_AA1487230; TRIAE_CS42_5DS_TGACv1_457109_AA1482480.2; TRIAE_CS42_5DS_TGACv1_457109_AA1482480.1 | NPR4F1-GCCCATCTGCGACCTCTCTGAC  NPR4R1- GGCCCACTCCGGTCGGGTGCCGCGC |

**^a^**The sequence of genes form *Arabidopsis* or Rice was used to identify wheat cv. Chinese Spring homologs via BLASTn in Ensembl Plants (*Triticum aestivum* (TGACv1, http://plants.ensembl.org/*Triticum_aestivum*).

**^b^**Primers were to target all listed homeologs. This was further validated in GSP: a web tool for genome specific primers design in polyploid species (<https://probes.pw.usda.gov/GSP/>).
