# Supplementary material for: A Pathogen-Responsive Leucine Rich Receptor Like Kinase Contributes to Fusarium Resistance in Cereals
Source: Front Plant Sci. 2018 Jun 26;9:867. doi: 10.3389/fpls.2018.00867 (PMC6029142; doi:10.3389/fpls.2018.00867)
Supplement: Supplementary file 5 [file Table_5.DOCX]

**Supplementary Table S5.** **Percent identity between homeologs from cv. Chinese Spring and chromosome 6D variants from cvs. CM82036 and Remus (based on deduced protein sequence).**

| **Gene variant : cultivar** | **TaLRRK-2A:Chinese spring** | **TaLRRK-2B:Chinese Spring** | **TaLRRK-2D:Chinese Spring** | **TaLRRK-6A:Chinese Spring** | **TaLRRK-6B:Chinese Spring** | **TaLRRK-6D:Chinese Spring** | **TaLRRK-6D:CM82036** | **TaLRRK-6D:Remus** |
| --- | --- | --- | --- | --- | --- | --- | --- | --- |
| **TaLRRK-2A:Chinese Spring** | 100.0 | 50.7 | 94.9 | 49.4 | 13.2 | 51.2 | 40.4 | 40.9 |
| **TaLRRK-2B:Chinese Spring** | 50.7 | 100.0 | 51.2 | 48.1 | 13.5 | 58.9 | 44.8 | 45.0 |
| **TaLRRK-2D:Chinese Spring** | 94.9 | 51.2 | 100.0 | 49.6 | 13.0 | 51.1 | 40.5 | 40.8 |
| **TaLRRK-6A:Chinese Spring** | 49.4 | 48.1 | 49.6 | 100.0 | 12.8 | 53.1 | 43.5 | 42.1 |
| **TaLRRK-6B:Chinese Spring** | 13.2 | 12.5 | 13.0 | 12.8 | 100.0 | 13.5 | 13.4 | 13.0 |
| **TaLRRK-6D:Chinese Spring** | 51.2 | 58.9 | 51.1 | 53.1 | 13.5 | 100.0 | 73.2 | 72.6 |
| **TaLRRK-6D:CM82036** | 40.4 | 44.8 | 40.5 | 43.5 | 13.4 | 73.2 | 100.0 | 88.1 |
| **TaLRRK-6D:Remus** | 40.9 | 45.0 | 40.8 | 42.1 | 13.0 | 72.6 | 88.1 | 100.0 |

*^a^*The sequence of *TaLRRK-6D* was used to identify wheat cv. Chinese Spring homologs via BLASTn in Ensembl Plants (*Triticum aestivum* (TGACv1, http://plants.ensembl.org/*Triticum_aestivum*). Then the deduced amino acids sequences of the homeologs and chromosome 6D variants from cvs. CM82036 and Remus variants were MAFFT aligned (Katoh et al., 2002) using Blusum62 matrix (Eddy, 2004) to measure the pairwise identity.
