# Supplementary material for: A Pathogen-Responsive Leucine Rich Receptor Like Kinase Contributes to Fusarium Resistance in Cereals
Source: Front Plant Sci. 2018 Jun 26;9:867. doi: 10.3389/fpls.2018.00867 (PMC6029142; doi:10.3389/fpls.2018.00867)
Supplement: Supplementary file 6 [file Data_Sheet_1.DOCX]

***Supplemental Results***

**A pathogen-responsive leucine rich receptor enhances cereal resistance to *Fusarium graminearum***

**Ganesh Thapa, Lokanadha Rao Gunupuru, James Gerard Hehir, Amal Kahla, Ewen Mullins, Fiona M Doohan ***

***Correspondence:** Fiona.doohan@ucd.ie

***In silico* analysis of the responsiveness of SA signaling genes to FHB disease**

The *Fusarium* responsiveness and expression profile of target *ICS1,* *NPR1, NPR3* and *NPR4* genes was analyzed using the Wheat Expression Browser (<http://www.wheat-expression.com/>), PLEXdb (Plant Expression Database), Expression Atlas (<https://www.ebi.ac.uk/gxa/about.html>) and the SRA database (<https://www.ncbi.nlm.nih.gov/sra)>. The *ICS1* gene targets (Supplemental Table S4) were not *Fusarium* responsive at 30h but were induced at 50 hours post inoculation (Kugler et al., 2013). The primers used to analyze *PAL* gene expression targeted six transcripts (Supplemental Table S5), all of which were *Fusarium* responsive at 30 and 48 and/or 50 hours post-inoculation (Kugler et al., 2013; Xiao et al., 2013). For *NPR1, NPR3* and *NPR4* gene targets (Supplemental Table S4)*,* expression was wither very low or not induced >1.5 fold by *Fusarium* at either 20 or 50hpi.
