# Supplementary material for: A Pathogen-Responsive Leucine Rich Receptor Like Kinase Contributes to Fusarium Resistance in Cereals
Source: Front Plant Sci. 2018 Jun 26;9:867. doi: 10.3389/fpls.2018.00867 (PMC6029142; doi:10.3389/fpls.2018.00867)
Supplement: Supplementary file 7 [file Image_1.PDF]

Supplementary Figures

A pathogen-responsive leucine rich receptor enhances cereal resistance to *Fusarium graminearum*

Ganesh Thapa, Lokanadha Rao Gunupuru, James Gerard Hehir, Amal Kahla, Ewen Mullins, Fiona M Doohan *

***Correspondence:** Fiona.doohan@ucd.ie

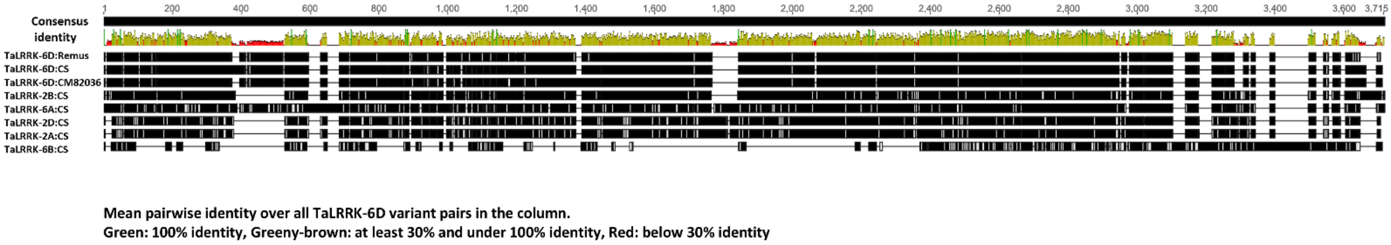

Supplementary Figure S1. Nucleotide alignment of TaLRRK-6D homeologs/variants from chromosome 6 and chromosome 2 of wheat cvs. Chinese spring (CS), and chromosome 6D variants fro, cvs. CM82036 and Remus. The nucleotide sequences were aligned with ClustalW (Thompson et al., 1997) cost matrix with cutoff gap open cost of 10 and gap extend cost of 0.1**.**

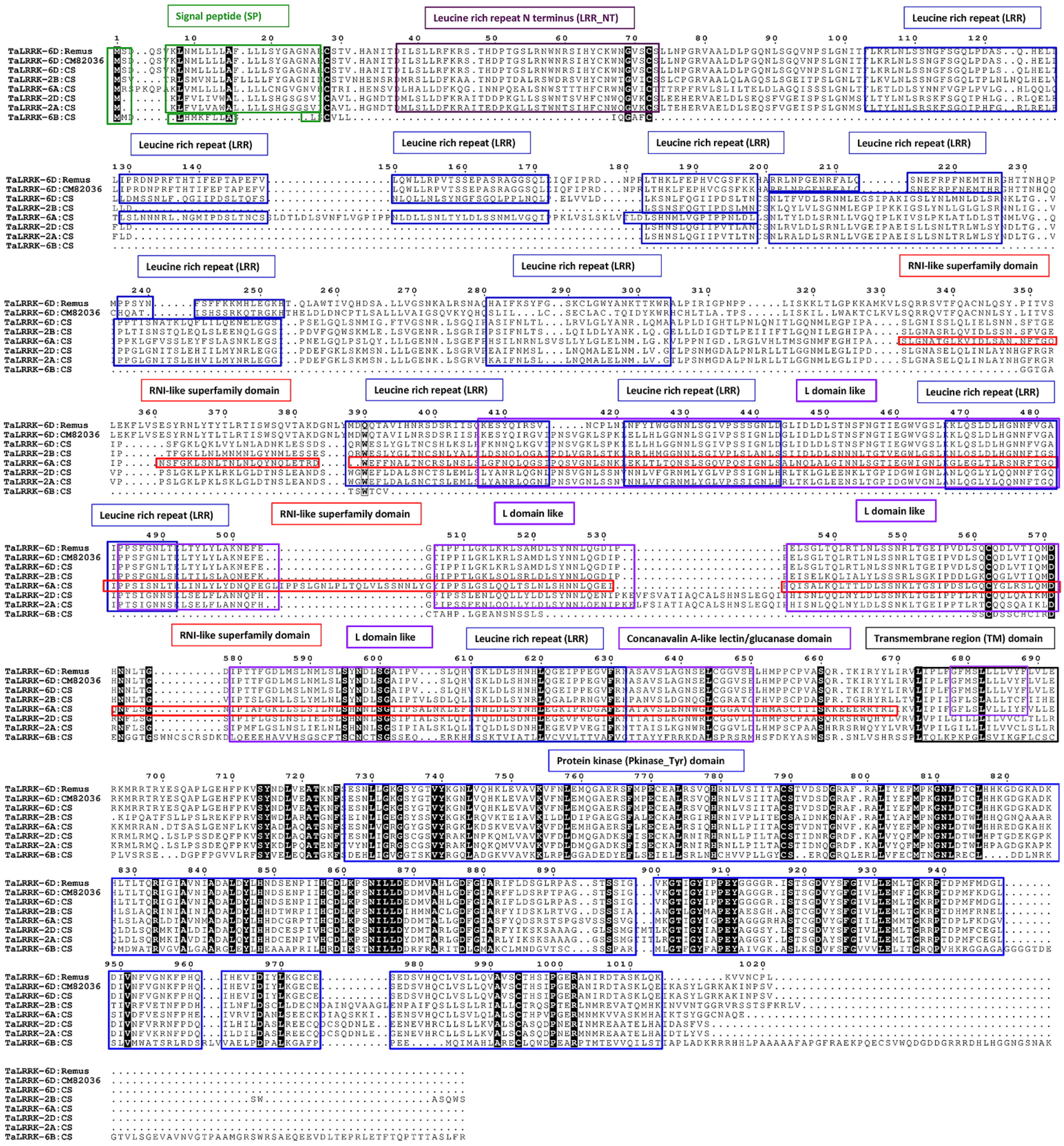

Supplementary Figure S2. Amino acids alignment showing the domains within the protein homologs and genotype variants of TaLRRK-6D. The amino acids sequences of the proteins were MAFFT (Katoh et al., 2002) aligned using BLUSUM62 (Eddy, 2004) cost matrix with gap open penalty of 1.53 and offset value.0.123 and percent identity calculated**.** The amino-acid sequences were scanned for conserved domains using the integrated InterPro protein database and domain/motif finding tools of all annotated plant, human and eukaryotic genes using Geneious R9 v.9.1.2 (Kearse et al., 2012). The TaLRRK-6D conserved domains were filtered and scanned through InterPro database (Mitchell et al., 2014). The deduced amino acid sequences contained the LRR-RLK conserved domains: signal peptide, LRR repeats, L domain like (IPR032675), concanavalin A-like lectin/glucanase domain (ID), TM domain, Protein kinase domain (ATP binding domain and Ser-threonine kinase domain). The conserved protein structure was then generated through Geneious protein structure builder.

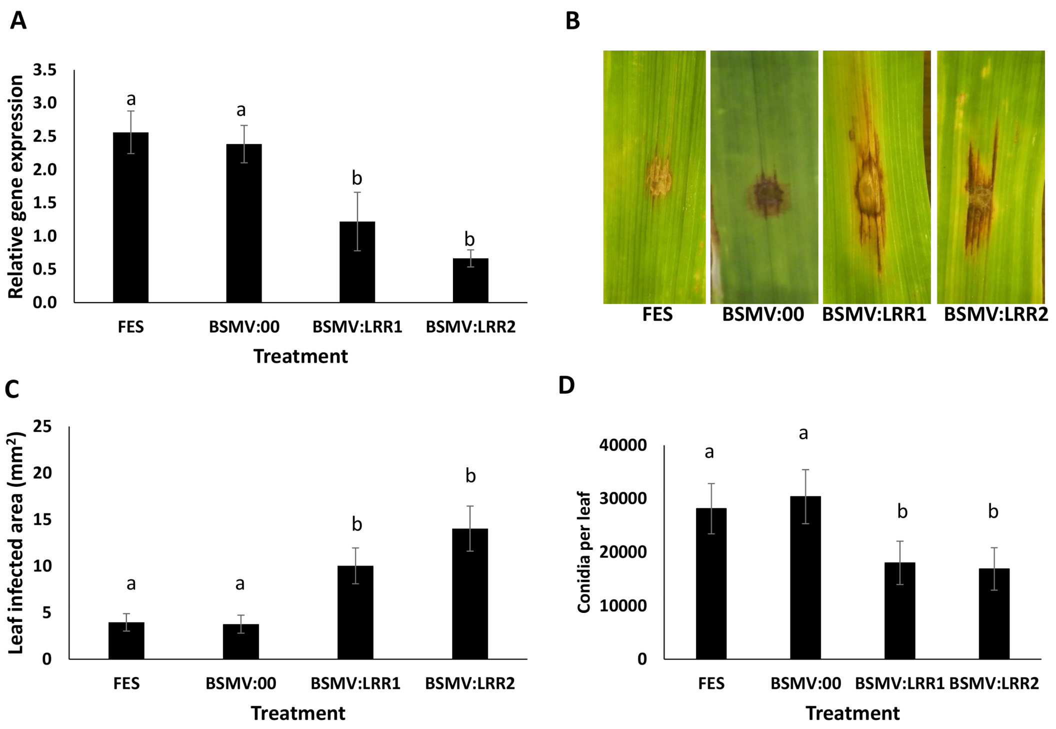

Supplementary Figure S3. Effect of virus induced gene silencing (VIGS) of *HvLRRK-6H* on the susceptibility of detached barley (cv. Akashinriki-uzu) leaves to *Fusarium culmorum*. The uzu derivative of cv. Akashinriki leaves were treated with either FES (the VIGS buffer), BSMV:00 (empty vector) or BSMV:LRR1 or BSMV:LRR2 (constructs targeting *HvLRRK-6H*). VIGS treatments was applied to the 2nd leaf and the 3rd leaf was detached and treated with a droplet of *F. culmorum* conidia. **(A)** Gene silencing of *HvLRRK-6H* in barley uzu leaves was quantified by real-time PCR analysis using reference genes barley actin (*HvActin*) and α-tubulin (*Hvα-tubulin*) and the 2^-ΔCt method (Livak and Schmittgen, 2001). **(B)** Symptoms of leaf necrosis four days post inoculation of spores on *HvLRRK-6H* silenced Akashinriki-uzu lines. **(C)** Quantification of area of infection using image J software in pixel count and converted to area of (2000 pixel = 0.1 cm^2). **(D)** Macroconidia production by *Fusarium* on the inoculated leaf segments. Results represent mean data obtained from 3 trials (n = 10 samples per treatment per time point) and bars in graphs indicate SEM. Treatments with the same letter are not significantly different (P> 0.05).

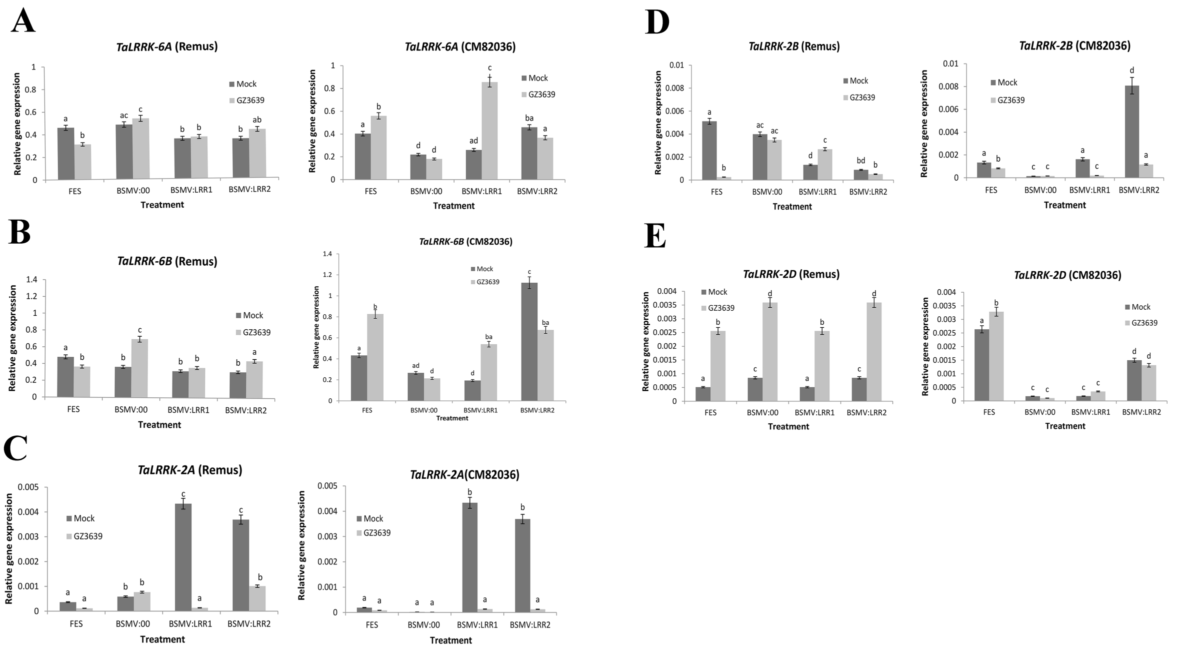

Supplementary Figure S4. Gene expression (qRT-PCR) analysis of chromosome 6A, 6B, 2A, 2B and 2D variants of *TaLRRK-6D* in plants treated with VIGS constructs targeting *TaLRRK-6D.* Plants of wheat cv. CM82036 and Remus were subjected to VIGS using barley stripe mosaic virus (BSMV) constructs. Plants were treated with either FES (the VIGS buffer), BSMV:00 (empty vector) or BSMV:LRR1 or BSMV:LRR2 (constructs targeting *TaLRRK-6D*). Flag leaves were treated with virus prior to emergence of the first head, and at mid anthesis (growth stage Zadoks 65) the two central florets of the spikelet were inoculated with either mock (Tween20) or conidia of wild type DON-producing *F. graminearum* strain GZ3639 or its DON-minus mutant derivative GZT40. The spikelet three above the treated spikelets was collected for gene expression studies. Gene silencing in wheat spikelets was quantified by real-time PCR analysis using the2^-ΔCt method (Livak and Schmittgen, 2001) to test for the silencing efficiency in TaLRRK-6A (**A**), *TaLRRK-6B* (**B**), *TaLRRK-2A* (**C**), *TaLRRK-2B* (**D**) and *TaLRRK-2D* (**E**) variants. Note the expression of the 2A, 2B and 2D variants is very low. Results represent mean data obtained from 2 trials (and in each, RNA was extracted from a bulk of 4 heads per treatment per time point and qRT-PCR was conducted twice per bulk RNA sample). Bars indicate SEM. Bars indicate SEM and treatments with same letter indicate are not significantly different (P > 0.05).

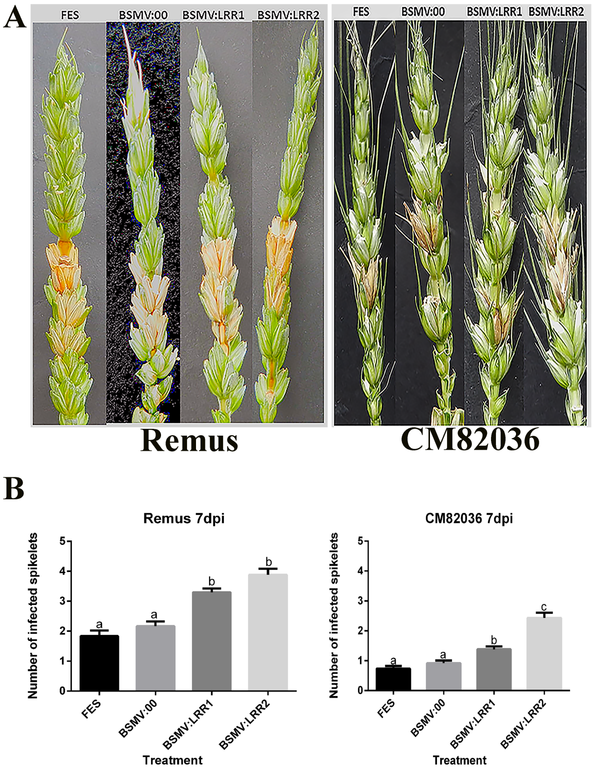

Supplementary Figure S5. Phenotypic effect of VIGS at 7dpi of *Fusarium* treatment. Disease symptoms was scored at 7 days post-*Fusarium* treatment. Plants of wheat cv. CM82036 and Remus were subjected to VIGS using barley stripe mosaic virus (BSMV) constructs. Plants were treated with either FES (the VIGS buffer), BSMV:00 (empty vector) or BSMV:LRR1 or BSMV:LRR2 (constructs targeting *TaLRRK-6D*).Flag leaves were treated with virus prior to emergence of first head, and at mid-anthesis (growth stage Zodaks 65) the two central florets of the spikelet were inoculated with either mock (Tween 20) or conidia of wild type DON-producing Fusarium graminearum GZ3639. **(A)** Wheat cvs. Remus and CM82036 spikelets displaying typical increased disease symptoms in *TaLRRK-6D* silenced lines. (**B**) Increased diseased spikelets in *TaLRRK-6D* silenced lines of cvs. Remus and CM82036. Disease and yield results represent mean data obtained from 60 heads (20 heads per treatment combination in each of three trials). Bars indicate SEM and treatments followed by the same letter are not significant different (P<0.05).

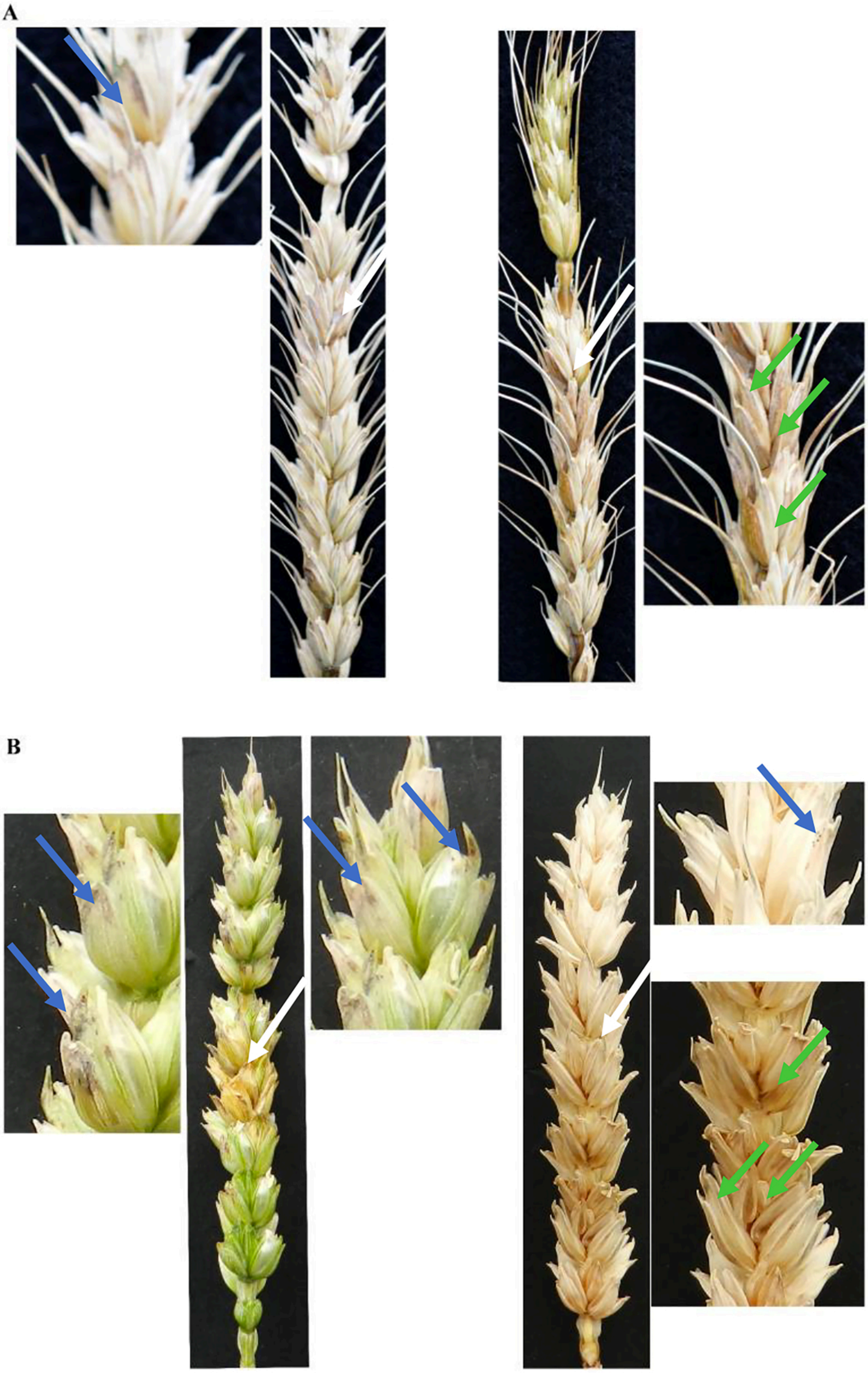

Supplementary Figure S6. Images of FHB symptoms on *TaLRRK-6D* silenced heads and close ups of spikelets from these heads for (A) cultivar CM82036 and (B) cultivar Remus at 21dpi of *Fusarium graminearum*. Plants of wheat cv. CM82036 and Remus were subjected to VIGS using barley stripe mosaic virus (BSMV) constructs. Images show those typical for plants treated with either the BSMV:LRR1 or BSMV:LRR2 (constructs targeting *TaLRRK-6D*) at 21 dpi. Flag leaves were treated with virus prior to emergence of first head, and at mid-anthesis (growth stage Zodaks 65) the two central florets of the spikelet were inoculated with wild type DON-producing *Fusarium graminearum* GZ3639. At this time point, the *TaLRRK-6D* silenced plants displayed maximal disease, with fungal growth visible on many spikelets. The point of inoculation, upward and downward fungal spread are respectively represented by white, blue and green arrows in the images.
